# Investigation of biochemical and physiological parameters of the newborn Saiga antelope (*Saiga tatarica*) in Gansu Province, China

**DOI:** 10.1371/journal.pone.0224822

**Published:** 2019-11-26

**Authors:** Xia Liu, James Blackar Mawolo, Xiaohua Du, Yingjie Zhou, Haifang Wang, Fayang Liu, Zhiqing He, Haqi Astika Marela

**Affiliations:** 1 Department of Animal Biotechnology, College of Life Science and Technology, Gansu Agricultural University, Lanzhou City, Gansu Province, People’s Republic of China; 2 Department of Basic Veterinary Medicine, College of Veterinary Medicine, Gansu Agricultural University, Lanzhou City, Gansu Province, People’s Republic of China; 3 Gansu Endangered Animal Protection Center of State Forestry Administration, Wuwei Town, Gansu Province, People’s Republic of China; University of Minnesota, UNITED STATES

## Abstract

**Background:**

The Saiga antelope (*Saiga tatarica*) is a critically endangered species, and there has been limited success in restoring the population by captive breeding. This study assessed the biochemical and physiological parameters of newborn Saiga antelope to provide reference information that can be used to evaluate their health. Comparisons have been made with parameters for horses and closely related members of the Bovidae family but there are no reference values for the newborn Saiga antelope.

**Methods:**

Biochemical and physiological parameters were measured in 61 animals. An automatic analyzer (Hitachi Ltd. 7180 Serial, Tokyo, Japan) was used to analyze the biochemical parameters, while the Coulter counter (Model ZK) was used to analyze the physiological parameters.

**Results:**

The results showed that the biochemical and physiological parameters differ considerably in range. The evaluation of parameters stratified by sex showed differences. Triglyceride and LDL cholesterol concentrations among male animals were significantly higher than those in female animals, while the creatine kinase concentrations were significantly higher in females than in males. Comparing this study’s results with published data for horses showed many similarities and some differences. Cholesterol, magnesium and glucose levels were similar between Saiga antelope and horses, while albumin and hematocrit levels in Saiga antelope differed from the reference values in horses.

**Conclusion:**

The study has shown that horses and even closely related members of the Bovidae family are not suitable references when evaluating the biochemical and physiological properties of newborn Saiga antelope. These animals have unique stressors and warrant further study to inform efforts pertaining to their care and the future sustainability of the species.

## Introduction

The Saiga antelope (*Saiga tatarica*) is a member of the family Bovidae and is native to Eurasia [1]. Since 1920, the animals have been protected. Frequent hunting of the Saiga antelope driven by the use of their horns in traditional Chinese medicine practices led to their near extinction [1]. In 2015, more than 200,000 Saiga antelope, representing half of the world’s Saiga antelope population, died from an outbreak of suspected pasteurellosis [2]. Despite significant concern regarding the preservation of the species, little information about newborn Saiga antelope is available.

For the assessment and monitoring of health, physiological and biochemical reference values have been established for a wide range of newborn animals. For example, reference values for hematological or biochemical parameters were established for Chital deer (*Axis*, *axis*) in Australia [3], Indian spiti horses [4], Southern chamois (*Rupicapra pyrenaica*) [5], Spanish ibex (*Capra pyrenaica*) [6], Black-faced impala (*Aepyceros melampus petersi*) [7], working horses [8], and some species of wild ruminants living in captivity [9]. In contrast, the available information to date on newborn Saiga antelopes has focused primarily on their feeding behavior. After the birth of Saiga antelope, they are fed by their mothers for up to four (4) months. In captivity, the newborn Saiga antelope has been observed nursing from adult Saiga antelope that are unrelated to them. The birthweight of the newborn Saiga antelope is on average 3.5 kg, and they grow to an average of 14.5 kg by the time they are weaned. The adults are herbivores and consume grasses, prostrate summer cypress, saltworts, sagebrush, and lichens.

In this study, the researchers present the biochemical and physiological parameters for male and female newborn Saiga antelopes to offer baseline metrics for the assessment of the animals’ health status. The results fulfilled the objective of this study.

## Materials and methods

### Animals and setting

A total of 61 male (n = 25) and female (n = 36) newborn Saiga antelope were used in this study. Only newborn Saiga antelope (1–3 days old) that showed no sign of any disease were included in this study. The samples were collected from animals at the Gansu Endangered Animals Research Center, located in Wuwei city of Gansu Province, China. Currently, over 109 Saiga antelopes are under observation at the center. The Gansu Endangered Animals Research Center was established in 1987 and covers a land area of 180,000 hectares. All animals involved in this study were from the Gansu Endangered Animals Research Center.

### Specimen sampling and preparation

The biochemical and physiological parameters were collected at separate times. The specimens were collected within a period of fifteen (15) days. All biochemical specimens were collected over the course of eight (8) days, while the physiological specimens were collected during another period of seven (7) days. Specimens were collected during the morning and evening hours. Animals were retrieved one at a time from their living area and immobilized to facilitate the drawing of blood from the jugular vein. The research center required immobilization of the animals to reduce complications due to sampling from the jugular vein. Therefore, per the guidance of resident veterinarians, this practice was undertaken to reduce harm to the animal subjects. Every effort was likewise made to reduce the number of animals used.

Blood samples were collected by trained personnel from the jugular vein of the animals into vacuette test tubes (6 ml) containing K_2_-EDTA and Na_2_. The biochemical analyses were conducted within a week after specimen collection using an automated analyzer (Hitachi Ltd. 7180 Serial, Tokyo, Japan) at the Animal Biochemistry Laboratory of Life Science and Technology College of Gansu Agricultural University. During the analysis of the biochemical parameters, the blood samples were centrifuged at 1000 rpm for ten (10) minutes. Serum and blood plasma were transferred into polypropylene microcentrifuge test tubes, which were stored at -80°C until analysis. After collecting the physiological parameters, the samples were checked within two (2) hours using a Coulter counter (Model ZK) at the Animal Biochemistry Laboratory of Life Science and Technology College of Gansu Agricultural University. All test tubes were sterilized before usage.

### Laboratory examinations

An automatic analyzer (Hitachi Ltd. 7180 Serial, Tokyo, Japan) was used to analyze the biochemical parameters, while a Coulter counter (Model ZK) was used to analyze the physiological parameters. Red and white blood cell counts were obtained using the Coulter counter. Red blood cells were stained with DAPI solution (Florida, USA). A total of 25 μl of blood was added to vials containing 4975 μl of DAPI solution (i.e., 200 × dilutions). Red blood cells were then counted in the chamber of the Coulter counter with the aid of a light microscope, while white blood cells were counted using Neubauer’s solution (Berlin’s Germany). A total of 25 μl of blood was added to vials containing 475 μl of Neubauer’s solution (i.e., 20 × dilutions). White blood cell differential counts were obtained from blood smears stained with Diff-Quick using an oil immersion objective and 1000x magnification. Determination of the lymphocyte count was performed using a complete blood count test, while the absolute neutrophil count (ANC) was performed to determine the level of granulocytes. Granulocytes were counted as part of the white blood cell (WBC) differential test.

Hematocrit levels were obtained following blood centrifugation in microcapillaries, while hemoglobin concentrations were measured using the Coulter method (England).

Biochemical parameters were determined spectrophotometrically. Urea and cholinesterase concentrations were determined using spectrophotometry, while the concentrations of glucose, total protein, HDLP, LDL cholesterol, alkaline phosphatase (ALP), and hydroxybutyrate dehydrogenase (HD) were measured using BioVendor (Czech Republic) tests. Quantitative serum tests were used to determine the level of immunoglobin. The concentration of albumin was determined using the microalbumin test (Beijing, People’s Republic of China). Phosphate tests (Human, Germany) were used for the determination of triglyceride and inorganic phosphorus concentrations, and the concentrations of minerals such as calcium and magnesium were determined using spectrophotometry.

### Ethics statement

The animal use protocol listed in this study was reviewed and approved by the Animal Ethical and Welfare Committee of Gansu Endangered Animals Research Center, Gansu Province in September 2018. Approval No. AEWC-GEARC-2018004.

### Collection and sampling method

The biochemical and physiological parameters were collected at separate times. The specimens were collected within a period of fifteen (15) days. All biochemical specimens were collected over the course of eight (8) days, while the physiological specimens were collected during another period of seven (7) days. Specimens were collected during the morning and evening hours. Animals were retrieved one at a time from their living area and immobilized to facilitate the drawing of blood from the jugular vein. The research center required immobilization of the animals to reduce complications due to sampling from the jugular vein. There was no method of sacrifice applied in this study. (This was already stated previously but per the email request, it is repeated here.)

### Details on animal disposition

The Gansu Endangered Animals Research Center provides a place for people to enjoy quality time during visitation and learn about animals while engaging in conserving wildlife and the natural environment. Since its establishment in 1987 by the State Forestry Administration of Gansu Province, the Gansu Endangered Animals Research Center has evolved over the years into a national and international conservation center with high standards of animal care, husbandry and treatment provided by a team of dedicated and professional staff. The Gansu Endangered Animals Research Center, China, has spearheaded wildlife conservation studies in China. With excellent care being the foundation of sustainable and responsible population management programs, the Center is also committed to raising industry standards by sharing its values and experience and supporting similar global initiatives. The Gansu Endangered Animals Research Center acquisition policy outlines its population management effort for whole-of-life care through its commitment to acquiring animals from similar responsible population management efforts. These include rescues, breeding loans, animal exchanges, and in some cases, purchases, from other accredited members of the Committee of Zoos and Aquariums (CZA) in Gansu Province. Animal acquisition from the wild is not undertaken without a thorough review of the circumstances, and in some cases, a population assessment would be required. A population assessment and nondetrimental findings report would precede the acquisition of any animal listed under appendices I, II or III of the Convention on International Trade in Endangered Species of Wild Fauna and Flora (CITES) or as “threatened” (defined as Critically Endangered, Endangered or Vulnerable) under the International Union for Conservation of Nature (IUCN) Red List. Gansu Endangered Animals Research Center’s policies for its program of responsible population management are stated within the CZA Code of Professional Ethics, a set of standards that guides all considerations of animal welfare matters within zoological facilities. In addition to this Code, an Institutional Program Animal Policy is in place to guide the manner in which Gansu Endangered Animals Research Center engages animals for programs, as well as an Institutional Animal Collection Plan to guide future decisions for the center’s zoological collection. All dispositions of animals at Gansu Endangered Animals Research Center must abide by the Mandatory Standards and General Advisories of the CZA Code of Professional Ethics; specifically, “a member shall make every effort to assure that all animals in his/her collection and under his/her care are disposed of in a manner which meets the current disposition standards of the Association and do not find their way into the hands of those not qualified to care for them properly.” Recipients of any live animals from the Gansu Endangered Animals Research Center are normally other accredited CZA members. However, in instances where this is not possible, a thorough review of the recipient facility by way of questionnaire and a site visit are conducted prior to the transfer. The aim of this review is to ensure that the recipient facility has the expertise, records management practices, financial stability, adequate facilities, and resources required to ensure positive animal welfare is maintained for the animals and their offspring. No animal disposition is permitted that could create a health or safety risk (to the animal or humans) or have a detrimental impact on the conservation of the species. If an animal is lost due to mortality, the cause of mortality will be thoroughly investigated and reviewed. All animal carcasses or samples not being stored for future research or ongoing pathology are disposed of in accordance with the health and safety regulations set by the Chinese government. Any death will be reported to the State Forestry Administration of Gansu Province, China for their records. Due to the proper care at the center, the death rate since the establishment of the center is minimal. A euthanasia policy is also in place in the event a living animal needs to undergo euthanasia. This policy follows the recommendations of the Chinese Veterinary Medical Association (CVMA) Guidelines for the Euthanasia of Animals. Euthanasia will generally be considered only after it is clear that appropriate alternatives such as treatment are unavailable or would be ineffective at preventing future suffering. Such consideration might be necessary for animals suffering due to disease, disability, injury or age-related factors where conditions cannot be resolved or alleviated. The Gansu Endangered Animals Research Center is one of the zoological facilities in China to report animal acquisitions and dispositions, including births and extremely low mortality data, in its annual report. The center’s data assurance has been further strengthened by engaging a third-party auditor.

### Animals housing conditions

The Gansu Endangered Animals Research Center is located in Wuwei city, Gansu Province, People’s Republic of China. The geographical coordinates are 37°52′45″N, 102°52′54″E, and the altitude is 1480 m. The center is located along the southern edge of the Tengger Desert, with a total area of 3900 hm^2^. The surface consists of moving dunes, fixed dunes, semifixed dunes and intermound depressions. The average number of sunny days each year is 73 (total annual hours of sunshine is 3246.7 hours). The average annual temperature is 7.7°C, the mean temperature of the hottest month (July) is 27.7°C and that of the coldest month (November) is -8.5°C. The annual precipitation is 110 ~ 170 mm, the annual evaporation is greater than 2020 mm, and the relative humidity is 40%. The temperature fluctuates between low and high (7–24°C), while the air quality is good. The Saiga antelope live in a 126.67 hm^2^ semiwild area. The animals live together (male and female) to enable reproduction. Saiga antelope are directed to the calving area before calving. The veterinarians monitor the feeding habitat and health status of the animals at the center. The animals are trained to cooperate with the veterinarians and investigative personnel. When it rains, the animals enter the chutes or cages with the aid of the center guides. The center is surrounded by a fence, and security measures are employed to safeguard the animals.

### Statistical analysis

The results are described as counts, means, standard deviations, and quantiles. Outliers were excluded using Grubbs’ test [10]. In addition, independent groups t-tests were applied to compare parameters between male and female antelope and to compare the study results with published data for Przewalski horses and domestic horses. Values of P<0.05 were considered statistically significant. Python analysis notebook was used.

## Results

The descriptive statistics for the biochemical and physiological parameters (Tables 1 and 2, respectively) from the overall sample of newborn Saiga antelope are different from those measured in closely related species. Compared to a study conducted on cattle [11], the hydroxybutyrate dehydrogenase and cholinesterase ranges in the Saiga antelope were higher. Magnesium and immunoglobin in the antelope had lower ranges than those reported in the Spanish ibex [9]. Other parameters, such as alkaline phosphatase and inorganic phosphorus, also had lower ranges when compared with published results on sheep [12]. Among the physiological parameters (Table 2), the level of hemoglobin was higher than the other parameters, such as lymphocytes and total red and white blood cells. Studies conducted on the Przewalski horse also reported a higher hemoglobin value and a lower lymphocyte value [13].

**Table 1:** Descriptive ranges of biochemical parameters.

Variables	COUNT	MEAN ± SD	MIN	MAX	Quantiles		
					25%	50%	75%
**Cholinesterase (x10^12^/L)**	89.00	345.72 **±** 151.56	-206.00	738.00	272.00	332.00	428.00
**Triglyceride (mmol/l)**	89.00	0.95 **±** 0.73	0.01	2.92	0.37	0.83	1.53
**Total Cholesterol (mmol/l)**	89.00	0.91**±** 0.39	0.00	1.83	0.66	0.86	1.14
**HDLP Cholesterol (mmol/l)**	00	0.29 **±** 0.16	-0.01	0.70	0.17	0.24	0.39
**LDLP Cholesterol (mmol/l)**	89.00	0.24 **±** 0.10	0.00	0.50	0.16	0.21	0.29
**Glucose (g/l)**	89.00	7.75 **±** 2.67	1.16	12.49	6.35	8.43	9.58
**Urea (mmol/l)**	89.00	8.13 **±** 2.88	-0.71	13.42	6.02	8.42	10.11
**Immunoglobin (g/l)**	89.00	0.15 **±** 0.13	0.00	0.58	0.06	0.13	0.24
**Total Protein (g/l)**	89.00	37.30 **±** 10.18	1.23	65.45	31.83	39.31	44.04
**Albumin (g/l)**	89.00	8.59 **±**7.90	4.08	1.97	6.14	1.16	1.55
**Alkaline Phosphatase (x10^6^/_mm_^3^_)_**	89.00	1.82 **±**7.29	-23.90	19.00	-2.00	1.00	7.00
**Hydroxybutyrate Dehydrogenase (U/l)**	89.00	628.95 **±** 150.66	19.14	907.00	531.00	661.00	738.00
**Calcium (mmol/l)**	89.00	1.66 **±** 0.12	1.20	1.97	1.60	1.64	1.77
**Magnesium (mmol/l)**	89.00	0.05 **±** 0.07	-0.11	0.22	0.03	0.07	0.10
**Inorganic Phosphorus (mmol/l)**	89.00	2.61 **±** 1.00	1.23	7.25	2.01	2.50	3.01

SD = standard deviation, MIN = minimum value, and MAX = maximum value

**Table 2:** Descriptive ranges of physiological parameters.

Variables	COUNT	MEAN ± SD	MIN	MAX	Quantiles		
					25%	50%	75%
**Total WBC (x10^9^/L)**	40.00	2.46 **±** 0.84	1.10	4.50	1.97	2.50	2.85
**Lymphocyte Ratio (%)**	00	18.59 **±** 3.89	12.10	29.80	16.27	17.95	20.70
**Intermediate (x10^9^/L)**	40.00	16.49 **±** 8.12	7.70	35, 100	11.17	13.30	18.07
**Granulocyte (%)**	40.00	64.90 **±** 10.93	42.20	79.10	59.52	67.65	71.90
**Hemoglobin (x10^9^/L)**	40.00	151.33 **±** 42.63	-22.00	535.00	276.50	334.00	424.75
**Hematocrit (x10^12^/L)**	40.00	73.59 **±** 13.32	0.01	2.80	0.21	0.46	1.24
**Total RBC (mmol/l)**	40.00	6.60 **±** 1.70	-0.01	0.70	0.17	0.19	0.43

SD = standard deviation, MIN = minimum value and MAX = maximum value

The biochemical parameters tended not to differ between male and female animals, with some exceptions (Table 3). On average, male newborn Saiga antelope had significantly higher levels of triglyceride and LDL cholesterol than female newborn Saiga antelope. In contrast, females had significantly higher levels of creatinine kinase than male animals (740.56 ± 348.729 vs 501.639 ± 225.249, p<0.001).

**Table 3:** Comparison of biochemical parameters for female and male newborn Saiga antelope.

	Female		Male		P-value
Variable	n	Mean ± SD	n	Mean ± SD	
**Cholinesterase (x10**^**12**^**/L)**	50	36.66 ±34.332	36	52.056 ± 58.931	0.1311
**Triglyceride (mmol/l)**	50	0.76 ± 0.483	36	1.238 ± 0.914	**0.0024
**Total Cholesterol (mmol/l)**	50	0.89 ± 0.345	36	1.027 ± 0.413	0.0888
**HDLP** **Cholesterol (mmol/l)**	50	0.27 ± 0.154	36	0.338 ± 0.176	0.0766
**LDLP Cholesterol (mmol/l)**	50	0.23 ± 0.095	36	0.276 ± 0.108	*0.0241
**Glucose (g/l)**	50	8.24 ± 2.156	36	7.446 ± 2.916	0.1522
**Urea (mmol/l)**	50	8.73 ± 2.693	36	7.964 ± 2.206	0.1628
**Immunoglobulin (g/l)**	50	0.15 ± 0.145	36	0.178 ± 0.127	0.3752
**Total protein (g/l)**	50	39.66 ± 16.621	36	39.218 ± 8.123	0.8818
**Albumin (g/l)**	50	24.60 ± 6.246	36	23.633 ± 6.676	0.4943
**Alkaline Phosphatase (IU/L)**	50	5.22 ±16.621	36	31.167 ± 136.371	0.1856
**Hydroxybutyrate Dehydrogenase (U/I)**	50	652.46 ± 196.890	36	631.806 ± 136.371	0.5895
**Creatine kinase (μmol/l)**	50	740.56 ± 348.729	36	501.639 ± 225.249	***0.00053
**Calcium (mmol/l)**	50	-1.67 ± 0.098	36	-1.673 ± 0.135	0.9476
**Magnesium (mmol/l)**	50	0.05 ± 0.084	36	-0.203 ± 1.681	0.2858
**Inorganic phosphorus (mmol/l)**	50	2.54 ± 1.141	36	2.629 ± 0.632	0.6707

SD = standard deviation values, n = number of samples, t-test P-value (

*p<0.05

**P<0.001

***P<0.0001)

Similarly, the physiological parameters generally did not vary significantly between male and female newborn Saiga antelope. Only the average red blood cell volume was significantly different, with females having a higher value than males in Table 4. (123.42 ± 3.80 vs 120.82 ± 3.28 g/l, P = 0.0277).

**Table 4:** Comparison of physiological/hematological parameters for female and male newborn Saiga antelope.

	Female		Male		P-Value
Variable	n	Mean ± SD	n	Mean ± SD	
**Total white blood cells (x10^9^/L)**	18	2.48 ± 0.91	21	2.38 ± 0.78	0.7067
**Lymphocyte ratio (%)**	18	19.51 ± 4.46	21	18.03 ± 3.19	0.2386
**Intermediate cell ratio (%)**	18	16.15 ± 8.12	21	17.21 ± 8.25	0.6894
**Granulocyte ratio (%)**	18	64.33 ± 10.12	21	64.76 ± 10.00	0.8963
**Lymphocyte (x10^9^/L)**	18	0.49 ± 0.24	21	0.41 ± 0.13	0.1915
**Intermediate cell (x10^9^/L)**	18	0.36 ± 0.17	21	0.39 ± 0.24	0.6628
**Granulocyte (%)**	18	1.63 ± 0.67	21	1.58 ± 0.60	0.7987
**Total red blood cells (x10^9^/L)**	18	6.60 ± 1.70	21	6.93 ± 1.16	0.4765
**Hemoglobin (x10^9^/L)**	18	151.33± 42.63	21	157.43± 30.51	0.6070
**Hematocrit (x10^12^/L)**	18	73.59 ± 13.32	21	79.49 ± 8.86	0.1073
**Average red blood cell volume (g/l)**	18	123.42 ± 3.80	21	120.82 ± 3.28	0.0277*
**Hemoglobin concentration (f/l)**	18	182.33 ± 8.11	21	184.76 ± 6.80	0.3154
**RBC distribution width SD (p/g)**	18	52.97 ± 3.09	21	53.00 ± 3.40	0.9754
**RBC distribution width CV (g/l)**	18	15.56 ± 0.69	21	15.84 ± 0.87	0.2667
**Total number of platelets (f/l)**	18	1089.72 ± 282.94	21	1203.14 ± 205.20	0.1563
**Average platelet volume (%)**	18	8.02 ± 0.46	21	8.29 ± 0.54	0.0986
**Platelet distribution width (x10^9^/L)**	18	5.02 ± 0.83	21	4.97 ± 0.34	0.7810
**Platelet pressure (x10^9^/L)**	18	0.88 ± 0.26	21	1.00 ± 0.19	0.1021
**Platelet large cell ratio (%)**	18	0.92 ± 0.17	21	0.99 ± 0.19	0.2461

SD = Standard deviation values, n = sample size, t-test P-value

(*p<0.05)

A comparison of newborn Saiga antelope parameters with published data on Przewalski and domestic horses is presented in Tables 5 and 6. The results presented here showed some similarities and differences compared to published data on horses [14], [10]. The cholesterol, magnesium and glucose levels were similar between Saiga antelope and horses, while albumin and hematocrit levels differed from the reported reference values in the horses.

**Table 5:** Comparison of newborn Saiga antelope biochemical parameters with published data on the horses.

Variables	Newborn Saiga antelope (n = 89)	*Equus przewalskii* (n = 20)	Ergebinese dei (n = 39–78)	*Equus caballus* (n = 4)	Light horse (n = 4)
**Total Cholesterol (mmol/l)**	0.91 ± 0.39	NA	2.44 ± 0.43	2.28 (2.18–2.36)	2.25 ± 0.34
**Glucose (g/l)**	7.75 ± 2.67	6.22 ± 1.17	8.27 ± 3.4	NA	4.55 ± 0.52
**Urea (mmol/l)**	8.13 ± 2.88	4.13 ± 2.75	5.92 ± 1.35	4.9–11.32	NA
**Total Protein (g/l)**	37.30 ± 10.18	69.0 ± 1.0	64.0 ± 6.0	71.0 (64–81)	64.0 ± 5.0
**Albumin (g/l)**	8.59 ± 7.90	NA	33.0 ± 3.0	36.0 (33–37)	NA
**Calcium (mmol/l)**	1.66 ± 0.12	3.0 ± 0.1	2.8 ± 0.23	2.38 (1.8–3.05)	2.98 ± 0.13
**Magnesium (mmol/l)**	0.05 ± 0.07	NA	NA	NA	0.82 ± 0.04

NA = not available, n = sample size

**Table 6:** Comparison of newborn Saiga antelope physiological parameters with published data on horses.

Variables	Newborn Saiga antelope (n = 40)	*Equus przewalskii* (n = 20)	Ergebinese dei (n = 39–78)	*Equus caballus* (n = 4)	Light horse (n = 44–55)
**Total WBC (x10^9^/L)**	2.46 ± 0.84	8.26 ± 1.68	8.3 ± 2.5	9.6 (8.4–10.8)	7.45 ± 1.28
**Lymphocytes (%)**	18.59 ± 3.89	2.8 ± 0.81	NA	NA	3.10 ± 0.75
**Hemoglobin** **(x10^9^/L)**	151.33 ± 42.63	155 ± 1.7	154 ± 2.0	163 (14.3–20)	146 ± 16.5
**Hematocrit (x10^12^/L)**	73.59 ± 13.32	43.7 ± 3.7	0.42 ± 0.07	0.47(0.38–0.50)	0.40 ± 0.04
**Total RBC (x10^9^/L)**	6.60 ± 1.70	8.9 ± 0.9	NA	8.6 (8.4–10.8)	8.8 ± 0.95

NA = not available, n = number of sample size

## Discussion

**Fig 1:**
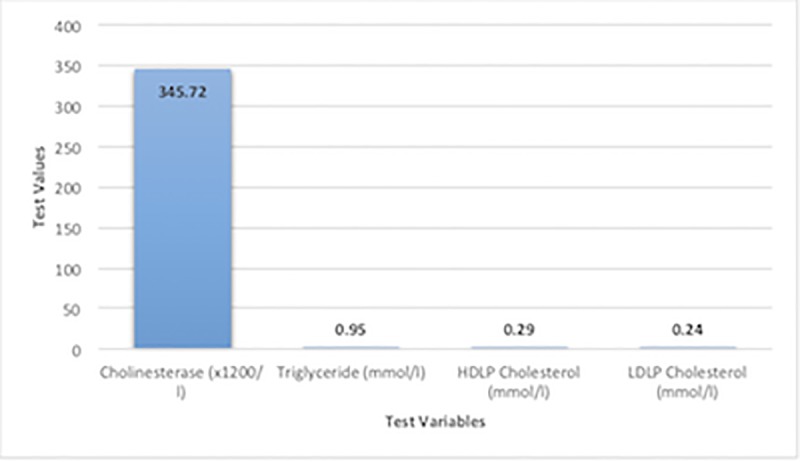
Comparison of closely related biochemical parameters.

**Fig 2:**
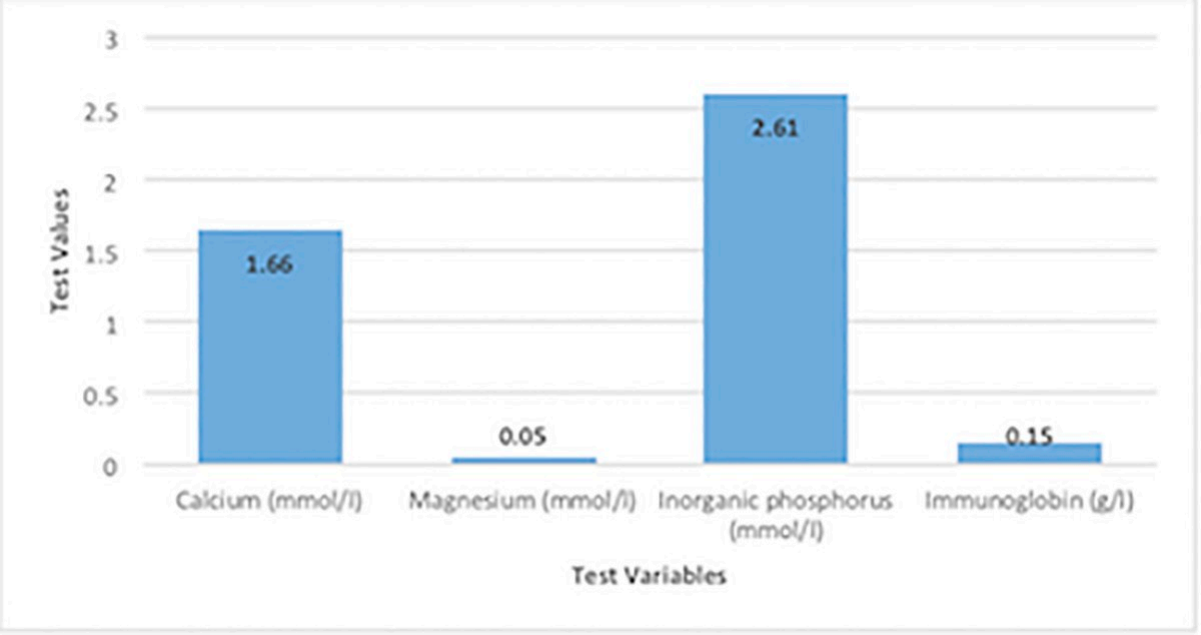
Comparison of more closely related biochemical parameters.

**Fig 3:**
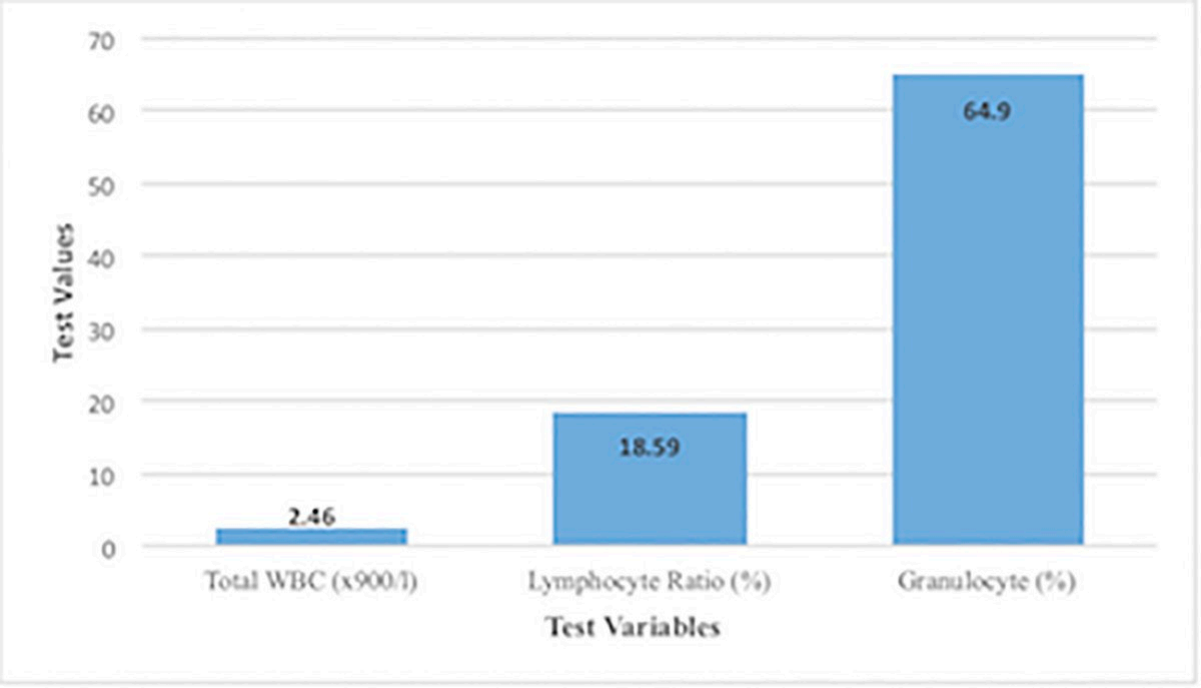
Comparison of closely related physiological parameters.

Parameters in the biochemical index were measured as the counts of 89, while the physiological parameters were measured as 40 counts. Research [9] conducted on the Spanish ibex showed that the concentrations of biochemical parameters were higher compared to those in newborn Saiga antelope. Studies [11],[12] performed on cattle and sheep reported different physiological parameter results compared to the results of this study. This might indicate the effect of blood sampling time on these parameters. The present findings in Tables 1 and 2 and Figs 1–3 might indicate a correlation between biochemical and physiological parameters in the newborn Saiga antelope. (Fig 1 compare closely related biochemical parameters while Fig 2 compare related biochemical parameters of close range and Fig 3 states the comparison among physiological parameters). However, these parameters were compared based on the ranges and close relationships and not statistically analyzed. These reports highlight the fact that results from one species may not necessarily be extrapolated to another, even if they are closely related. Future work on the comparability of results across species will be important, especially with regard to removing potential confounding factors, such as blood sampling time.

### Differences in biochemical parameters by sex

On average, male newborn Saiga antelope had significantly higher triglyceride concentrations (1.238 ± 0.914 mmol/l) than females (0.76 ± 0.483 mmol/l). The results in Turkmen horses or Akhal-Teke male triglyceride levels were higher than the females levels, although the result was not significantly different [1]. The average LDL cholesterol level was significantly higher in males (0.276 ± 0.108 mmol/l) than in females (0.23 ± 0.095 mmol/l). This result is contrary to published data on the Przewalski horse, which reported that the average LDL cholesterol was higher in females than in males, although the difference was not significant [13]. The findings are more consistent with published data about the Turkmen horse, which reported that concentrations of LDL cholesterol tended to be higher in males than in females, although the difference was not significant [1]. In the Saiga antelope, the creatine kinase concentration was significantly higher in females (740.56 ± 348.729 μmol/l) than in males (501.639 ± 225.249 μmol/l). The creatine kinase concentrations were significantly higher in both sexes of Przewalski horses than in antelope [13]. In newborn Saiga antelope, the concentrations of HDLP cholesterol (P = 0.07) and immunoglobin G (P = 0.37) were higher values in females than in males, although the difference was not significant. The concentration of magnesium (P = 0.28) in this study was higher in males than in females, although the difference was not statistically significant, and the trend in magnesium levels was similar in Przewalski horses [13].

A study on the Przewalski horse [13] reported a higher value of inorganic phosphorus in younger individuals than in older individuals, and showed that the concentration of inorganic phosphorus was higher in males (2.629 ± 0.632 mmol/l) than in females (2.54 ± 1.141 mmol/l). This phenomenon is associated with growth and higher bone metabolism. The glucose concentration was higher in females (8.24 ± 2.156 g/l) than in males (7.446 ± 2.916 g/l), although the difference was not statistically significant, while other studies [15] have shown glucose to have the most profound difference, with a nearly two-fold difference between the sexes in domestic horses [10]. The urea concentration was higher in females (8.73 ± 2.693 mmol/l) than in males (7.964 ± 2.206 mmol/l), but a study [16] on the *Ergebinese dei* reported the opposite results. The urea concentration in the *Ergebinese dei* was lower in females than in males. The male and female concentrations of albumin, total protein, and hydroxybutyrate dehydrogenase did not differ from most references. These results are similar to the reference values for Przewalski and Turkmen horses [1], [13], which reported normal reference ranges for these parameters.

### Differences in physiological parameters by sex

An evaluation was made of the sex-based differences. The average red blood cell concentration was significantly higher in females (123.42 ± 3.80 x10^9^/L) than in males (120.82 ± 3.28 x10^9^/L). This result differs from the finding in the study [13] conducted on the Przewalski horse, which showed that the average red blood cell concentration was significantly higher in the males than in the females. In this study reported a higher value was found for males (64.76 ± 10.00%) than females (64.33 ± 10.12%) with respect to granulocytes. A study on the Przewalski horse showed similar results, i.e., the granulocyte concentration was significantly higher in males than in females [13]. The concentration of granulocytes was also significantly higher in males than in females in light horses [10]. In addition, another study [17] has shown that young individuals have significantly higher concentrations of granulocytes than adult individuals. Hemoglobin, reported for the Przewalski horse, was significantly higher in males than in females, but this study showed that in the Saiga antelope, the concentration of hemoglobin was nonsignificantly higher in males (184.76±6.80 x10^9^/L) than in females (182.33± 8.11 x10^9^/L). In this study, the average WBC concentration was higher in females (2.48 ± 0.9 x10^9^/L) than in males (2.38 ± 0.78 x10^9^/L), but a study [13] conducted on the Przewalski horse showed that the male WBC concentration was higher than the female WBC concentration. Domestic horses also had higher concentrations of WBCs in males than in females, although the difference was not significant. The average concentration of hematocrit was higher in males (79.49 ± 8.86 x10^12^/L) than in females, although the difference was not significant. A study on Indian Spiti horses [4] showed that hematocrit concentration decreased with age but that the trend was not significantly difference. In this study, the concentration of lymphocytes was higher in females (19.51 ± 4.46 x10^9^/L) than in males (18.03 ± 3.19 x10^9^/L)_._ A study on the Przewalski horse [13] observed no differences in lymphocyte concentration between males and females. Other parameters in males and females had lower values compared to published results on horses. Data concerning biochemical and physiological values are still limited, and thus far, this study is one of the most extensive studies on the Saiga antelope, especially the newborn Saiga antelope.

### Comparison of present study results with published results

There exist few references for biochemical and physiological parameters, and comparing the results of this study with published data on *Equus przewalskii* and *E*. *caballus* resulted in the identification of notable differences. This study reported that the level of albumin in the newborn Saiga antelope differed from reported reference values in *E*. *przewalskii* and *E*. *caballus*. The hematocrit result in this study was higher than the reference values reported for *Equus przewalskii* and *E*. *caballus*. Other examined parameters, such as the levels of glucose, urea, total cholesterol, calcium, and magnesium, when compared between the Saiga antelope and both *Equus przewalskii* and *E*. *caballus* were similar; the values were lower in the Saiga antelope, but they were within the same ranges. Another study conducted on the Spiti horses from India [4] showed an opposite trend, i.e., the level of urea was higher than the reference range. Levels of urea can increase during stressful situations due to muscular activity and renal vasospasm produced by catecholamines [18], [19]. The urea value present in the newborn Saiga antelope in this study is similar to the baseline value found [20] in lambs subjected to different diets. The total protein range in this study corresponded with that in the study [15] conducted on *E*. *przewalskii*. Parameters with values lower than the references may have been the result of the from age at blood sampling, the timely collection of specimens, and the higher levels of stress in animals not accustomed to daily handling [21]. The levels of lymphocytes, WBC, and RBC, when compared with published data on *Equus przewalskii* and *E*. *caballus*, were similar, although low in the reference range, while the level of hemoglobin was high in the reference range. The higher level of hemoglobin in this study was contrary to the results of studies conducted on domestic horses. Studies [15], [16] reported that there was a significant drop in hemoglobin concentration during the course of anesthesia. Similar differences to those seen in the Przewalski horses were reported for feral horses [22]. The researchers recognize that the negative values obtained for calcium and magnesium are a study limitation.

## Conclusion

The study results show certain differences and similarities in the biochemical and physiological parameters in newborn Saiga antelope compared to horses and members of the Bovidae family. The available references for the horses and closely related members of the Bovidae family are not suitable when evaluating biochemical and physiological specimens of the newborn Saiga antelope. It is not possible to exclude the effect of stress on the physiological parameters, and it is almost impossible to exclude subclinical diseases in animals living in a particular area. The animals are living under different conditions and climatic areas; therefore, their feeding habits vary. All of these factors, among others, can influence the measured ranges of parameters. This study recommends that researchers collect specimens from animals across species living under comparable conditions. With regard to the sample size and the age of the Saiga antelope, this study is unique.

## Supporting information

S1 File Summary of figures. (PDF)

S2 File Analysis of biochemical data. (PDF)

S3 File Rerun analysis of biochemical data. (PDF)

S4 File Analysis of physiological data. (PDF)

S5 File Rerun analysis of physiological data. (PDF)

S6 File Laboratory protocols DOIs: dx.doi.org/10.17504/protocols.io.6wchfaw and dx.doi.org/10.17504/protocols.io.6wehfbe. (PDF)

S7 File Analysis of biochemical data. (PDF)

S8 File Analysis of physiological data. (PDF)
