# Correction: Investigation of biochemical and physiological parameters of the newborn Saiga antelope (*Saiga tatarica*) in Gansu Province, China

**DOI:** 10.1371/journal.pone.0230095

**Published:** 2020-03-02

**Authors:** Xia Liu, James Blackar Mawolo, Xiaohua Du, Yingjie Zhou, Haifang Wang, Fayang Liu, Zhiqing He, Haqi Astika Marela

The first and second authors are incorrectly listed as having contributed equally to this work. The correct byline is: Xia Liu, James Blackar Mawolo, Xiaohua Du, Yingjie Zhou, Haifang Wang, Fayang Liu, Zhiqing He, Haqi Astika Marela.
