# Supplementary material for: Investigation of biochemical and physiological parameters of the newborn Saiga antelope (Saiga tatarica) in Gansu Province, China
Source: PLoS One. 2019 Nov 26;14(11):e0224822. doi: 10.1371/journal.pone.0224822 (PMC6879164; doi:10.1371/journal.pone.0224822)
Supplement: S1 File — (PDF) [file pone.0224822.s001.pdf]

Table1: Descriptive range of biochemical parameters

**Variables**	COUNT	MEAN ± SD	MIN	MAX	Quantities of percentages		
					**25%**	**50%**	**75%**
**Cholinesterase ($x10^{12}$/l)**	89.00	345.72 ± 151.56	-206.00	738.00	272.00	332.00	428.00
**Triglyceride (mmol/l)**	89.00	0.95 ± 0.73	0.01	2.92	0.37	0.83	1.53
**Total Cholesterol (mmol/l)**	89.00	0.91± 0.39	0.00	1.83	0.66	0.86	1.14
**HDLP Cholesterol (mmol/l)**	89. 00	0.29 ± 0.16	-0.01	0.70	0.17	0.24	0.39
**LDLP Cholesterol (mmol/l)**	89.00	0.24 ± 0.10	0.00	0.50	0.16	0.21	0.29
**Glucose (g/l)**	89.00	7.75 ± 2.67	1.16	12.49	6.35	8.43	9.58
**Urea (mmol/l)**	89.00	8.13 ± 2.88	-0.71	13.42	6.02	8.42	10.11
**Immunoglobin (g/l)**	89.00	0.15 ± 0.13	0.00	0.58	0.06	0.13	0.24
**Total Protein (g/l)**	89.00	37.30 ± 10.18	1.23	65.45	31.83	39.31	44.04
**Albumin (g/l)**	89.00	8.59 ±7.90	4.08	1.97	6.14	1.16	1.55
**Alkaline Phosphatase ($x10^{6}/_{mm^3}$)**	89.00	1.82 ±7.29	-23.90	19.00	-2.00	1.00	7.00
**Hydroxybutyrate dehydrogensae (U/l)**	89.00	628.95 ± 150.66	19.14	907.00	531.00	661.00	738.00
**Calcium (mmo/l)**	89.00	1.66 ± 0.12	1.20	1.97	1.60	1.64	1.77
**Magnesium (mmo/l)**	89.00	0.05 ± 0.07	-0.11	0.22	0.03	0.07	0.10
**Inorganic phosphorus (mmo/l)**	89.00	2.61 ± 1.00	1.23	7.25	2.01	2.50	3.01

- SD= standard deviation, minimum and maximum values.

Table 2: Descriptive ranges of physiological parameters

**Variables**	**COUNT**	**MEAN ± SD**	**MIN**	**MAX**	Quantities of percentages		
					**25%**	**50%**	**75%**
**Total WBC ($x10^{9/}{}_{1)}$**	40.00	2.46 ± 0.84	1.10	4.50	1.97	2.50	2.85
**Lymphocyte Ratio ($x10^{9/}{}_{1)}$**	40. 00	18.59 ± 3.89	12.10	29.80	16.27	17.95	20.70
**Intermediate ($x10^{9/}{}_{1)}$**	40.00	16.49 ± 8.12	7.70	35, 100	11.17	13.30	18.07
**Granulocyte ($x10^{9/}{}_{1)}$**	40.00	64.90 ± 10.93	42.20	79.10	59.52	67.65	71.90
**Cholinesterate ($x10^{12}$/l)**	40.00	338.29 ± 111.66	-22.00	535.00	276.50	334.00	424.75
**Triglyceride (mmol/l)**	40.00	0.79 ± 0.74	0.01	2.80	0.21	0.46	1.24

**Total Cholesterol (mmol/l)**	40.00	0.90 ± 0.46	0.00	1.83	0.59	0.72	1.25
**HDLP Cholesterol (mmol/l)**	40.00	0.28 ± 0.18	-0.01	0.70	0.17	0.19	0.43

SD= standard deviation, minimum and maximum values.

Table 3. Comparison of biochemical parameters of female and male

	**Female**		**Male**		**P-value**
**Variable**	**n**	**Mean ± SD**	**n**	**Mean ± SD**	
**Cholinesterase (x10^{12}/l)**	50	36.66 ±34.332	36	52.056 ± 58.931	0.1311
**Triglyceride (mmol/l)**	50	0.76 ± 0.483	36	1.238 ± 0.914	****0.0024**
**Total cholesterol (mmol/l)**	50	0.89 ± 0.345	36	1.027 ± 0.413	0.0888
**HDLP Cholesterol (mmol/l)**	50	0.27 ± 0.154	36	0.338 ± 0.176	0.0766
**LDLP Cholesterol (mmol/l)**	50	0.23 ± 0.095	36	0.276 ± 0.108	***0.0241**
**Glucose (g/l)**	50	8.24 ± 2.156	36	7.446 ± 2.916	0.1522
**Urea (mmol/l)**	50	8.73 ± 2.693	36	7.964 ± 2.206	0.1628
**Immunoglobulin (g/l)**	50	0.15 ± 0.145	36	0.178 ± 0.127	0.3752
**Total protein (g/l)**	50	39.66 ± 16.621	36	39.218 ± 8.123	0.8818
**Albumin (g/l)**	50	24.60 ± 6.246	36	23.633 ± 6.676	0.4943
**Alkaline phosphatase (x10^{6}/mm^{3})**	50	5.22 ±16.621	36	31.167 ± 136.371	0.1856
**Hydroxybutyrate dehydrogensae (U/l)**	50	652.46 ± 196.890	36	631.806 ± 136.371	0.5895
**Creatine kinase (umol/l)**	50	740.56 ± 348.729	36	501.639 ± 225.249	*****0.00053**
**Calcium (mmo/l)**	50	-1.67 ± 0.098	36	-1.673 ± 0.135	0.9476
**Magnesium (mmo/l)**	50	0.05 ± 0.084	36	-0.203 ± 1.681	0.2858
**Inorganic phosphorous (mmol/l)**	50	2.54 ± 1.141	36	2.629 ± 0.632	0.6707

SD=standard deviation values, n=number of samples, t-test P-value (*p<0.05, **P<0.001, ***P<0.0001)

Table 4. Comparison of physiological/hematological parameters of female and male

	**Female**		**Male**		**P-Value**
**Variable**	**n**	**Mean ± SD**	**n**	**Mean ± SD**	
**Total white blood cells (x10^{9}/1)**	18	2.48 ± 0.91	21	2.38±0.78	0.7067

**Lymphocyte ratio ($x10^9$/l)**	18	19.51 ± 4.46	21	18.03±3.19	0.2386
**Intermediate cell ratio ($x10^9$/l)**	18	16.15 ± 8.12	21	17.21±8.25	0.6894
**Granulocyte ratio ($x10^9$/l)**	18	64.33± 10.12	21	64.76±10.00	0.8963
**Lymphocyte ($x10^9$/l)**	18	0.49 ± 0.24	21	0.41±0.13	0.1915
**Intermediate cell ($x10^9$/l)**	18	0.36 ± 0.17	21	0.39 ±0.24	0.6628
**Granulocyte ($x10^9$/l)**	18	1.63 ± 0.67	21	1.58±0.60	0.7987
**Total Number of red blood cells ($x10^{12}$/l)**	18	6.60 ± 1.70	21	6.93±1.16	0.4765
**Hemoglobin (g/l)**	18	151.33±42.63	21	157.43±30.51	0.6070
**Hematocrit (%)**	18	73.59± 13.32	21	79.49±8.86	0.1073
**Average red blood cell volume (10^{12}/mm^3)**	18	123.42± 3.80	21	120.82±3.28	**0.0277***
**Hemoglobin content (g/l)**	18	22.74 ± 1.03	21	22.59 ±0.88	0.6293
**Hemoglobin concentration (g/l)**	18	182.33± 8.11	21	184.76±6.80	0.3154
**Red blood cell distribution width SD ($x10^{12}$/l)**	18	52.97 ± 3.09	21	53.00±3.40	0.9754
**Red blood cell distribution width CV($x10^{12}$/l)**	18	15.56 ± 0.69	21	15.84±0.87	0.2667
**Total number of platelets ($x10^9$/l)**	18	1089.72±282.94	21	1203.14±205.20	0.1563
**Average platelet volume ($x10^9$/l)**	18	8.02 ± 0.46	21	8.29±0.54	0.0986
**Platelet distribution width ($x10^9$/l)**	18	5.02 ± 0.83	21	4.97 ±0.34	0.7810
**Platelet pressure ($x10^9$/l)**	18	0.88 ± 0.26	21	1.00 ±0.19	0.1021
**Platelet large cell ratio ($x10^9$/l)**	18	0.92 ± 0.17	21	0.99 ±0.19	0.2461

SD=Standard deviation values, n=samples size, t-test P-value (*$p<0.05$)

Table 5: Comparison of biochemical parameters of Newborn Saiga antelope with published data on the horses.

**Variables**	**Newborn Saiga antelope (n=36)**	**Equus Przewalski** (n=20)	**Ergebinese dei** (n=39-78)	**Equus horse** (n=4)	**Light horse** (n=4)
**Total protein (g/l)**	39.218 ± 8.123	69 ± 1	64 ± 6	71 (64-81)	64 ± 5
**Albumin (g/l)**	23.633 ± 6.676	NA	33 ± 3	36 (33-37)	31 ± 2
**Glucose (g/l)**	7.446 ± 2.916	6.22 ± 1.17	8.27± 3.4	NA	4.55±0.52
**Creatine kinase (umol/l)**	501.639 ± 225.249	99.01 ± 25.64	106.01 ± 17.68	141.44 - 265.2	115 ± 18
**Urea (mmol/l)**	7.446 ± 2.916	4.13 ± 2.75	5.92 ± 1.35	4.9 - 11.32	NA
**Total Cholesterol (mmol/l)**	1.027 ± 0.413	NA	2.44 ± 0.43	2.28(2.18-2.36)	2.25 ± 0.34
**Calcium (mmol/l)**	-1.673 ± 0.135	3 ± 0.1	2.8 ± 0.23	2.38 (1.8-3.05)	2.98 ± 0.13
**Magnesium (mmol/l)**	-0.203 ± 1.681	NA	NA	NA	0.82±0.04

NA=not available, n=samples size

Table 6: Comparison of physiological parameters of Newborn Saiga antelope with published data on the horses.

Variables	Newborn Saiga antelope (n=18)	Equus Przewalski (n=20)	Ergebinese dei (n=39-78)	Equus horse (n=4)	Light horse (n=44-55)
**Hemoglobin (g/1)**	151.33 ± 42.63	155 ± 1.7	154 ± 2	163 (14.3–20)	146 ± 16.5
**Lymphocytes (x10/1)**	0.49 ± 0.24	2.8 ± 0.81	NA	NA	3.10 ± 0.75
**Hematocrit (1/1)**	73.59 ± 13.32	43.7 ± 3.7	0.42 ± 0.07	0.47(0.38–0.50)	0.40 ± 0.04
**Total WBC (x10/1)**	2.48 ± 0.91	8.26 ± 1.68	8.3 ± 2.5	9.6 (8.4–10.8)	7.45 ± 1.28
**Total RBC (x10/1)**	6.60 ± 1.70	8.9 ± 0.9	NA	8.6 (8.4–10.8)	8.8 ± 0.95

NA=not available, n=samples size

Fig 1 below compares range of closely related parameters of Triglyceride, Cholinesterate, HDLP and LDLP

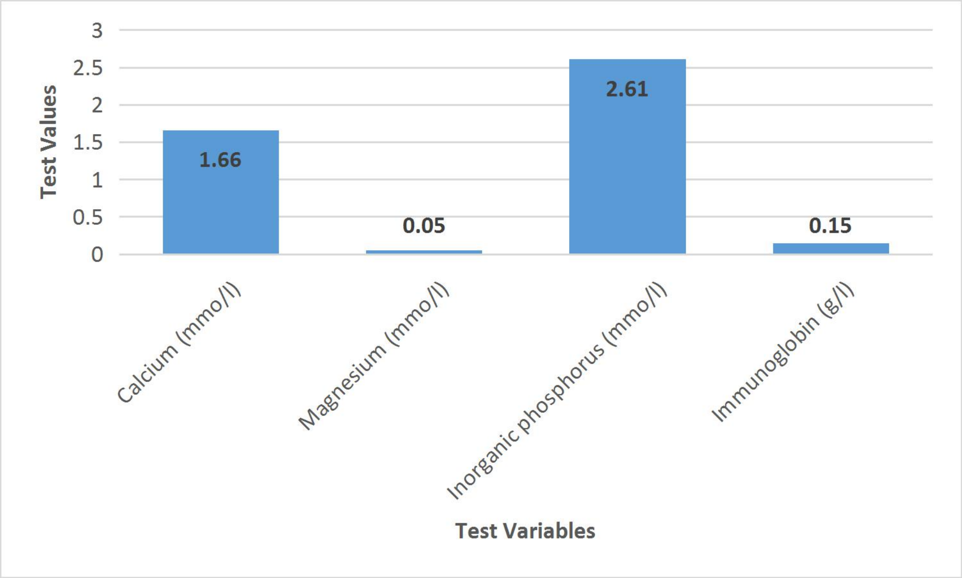

Fig 1: Comparison of closely related biochemical parameters

Fig 2. below sets out the comparison among parameters of calcium, magnesium, phosphorous and immunoglobin.

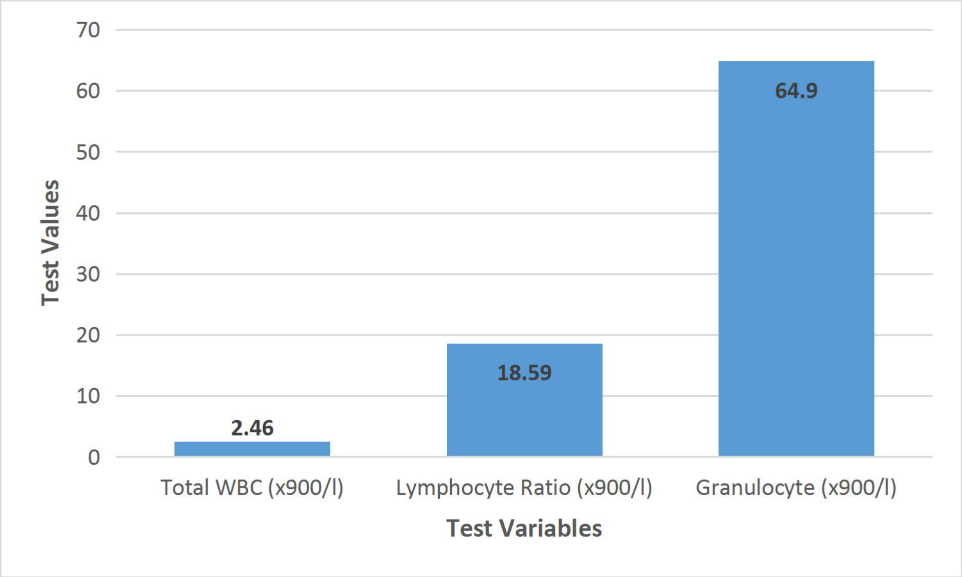

Fig 2: Comparison of closely related biochemical parameters

Fig 3. explains comparison among parameters of total WBC, lymphocyte ratio and granulocyte. All these have been compared base on ranges but not statistically.

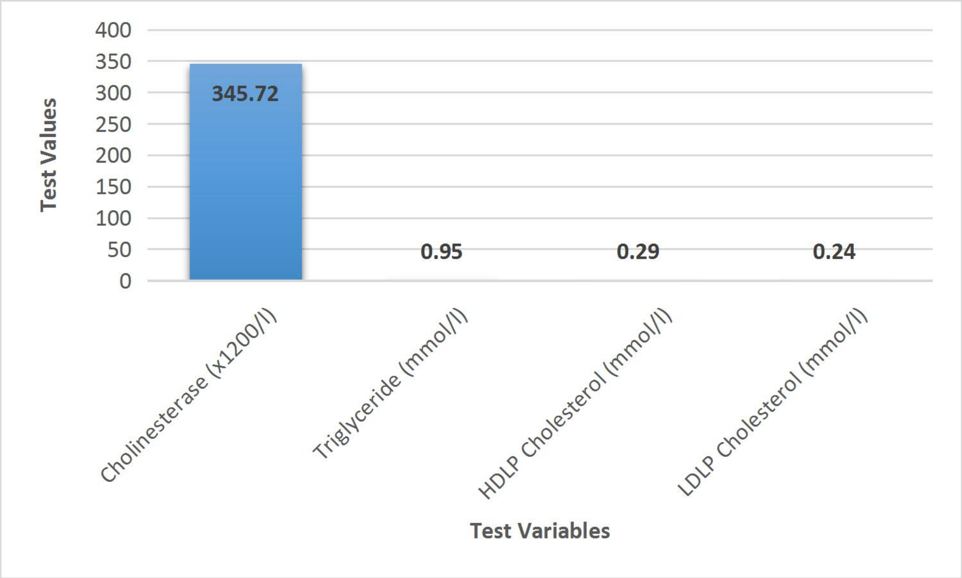

Fig 3: Comparison of closley related physiological parameters
