# Supplementary material for: Investigation of biochemical and physiological parameters of the newborn Saiga antelope (Saiga tatarica) in Gansu Province, China
Source: PLoS One. 2019 Nov 26;14(11):e0224822. doi: 10.1371/journal.pone.0224822 (PMC6879164; doi:10.1371/journal.pone.0224822)
Supplement: S2 File — (PDF) [file pone.0224822.s002.pdf]

Python Analysis Notebook

November 18, 2018

```
In [1]: #import the libreries for the analysis
        import pandas as pd
        import numpy as np
        import matplotlib.pyplot as plt
        %matplotlib inline

In [4]: #read in the data from the csv file
        df=pd.read_csv("mawoloCleanData.csv")
        #view the first five rows from the dataset
        df.head()

Out[4]:    cholinesterase  triglyceride  totalcholesterol  \
        0           266.0          1.16              1.83
        1           254.0          1.14              1.83
        2           214.0          1.48              1.77
        3           280.0          1.48              1.80
        4           535.0          0.33              0.60

           highdensitylipoproteincholestero  lowdensitylipoproteincholesterol  \
        0                              0.69                              0.48
        1                              0.70                              0.48
        2                              0.62                              0.48
        3                              0.64                              0.50
        4                              0.17                              0.16

           glucose  urea  immunoglobuling  totalprotein       albumin  \
        0     8.46  5.24             0.08     42.630001  4.080186e-07
        1     8.29  5.32             0.41     42.860001  2.040093e-06
        2     8.65  4.59             0.00     43.240002  6.097965e-06
        3     8.94  4.70             0.36     45.830002  1.421690e-05
        4    10.16  8.39             0.44     33.820000  2.841626e-05

           alkalinephosphatase  hydroxybutyratedehydrogenase  creatinekinase  calcium  \
        0             1.157303                         784.0           686.0     1.74
        1             2.134831                         774.0           694.0     1.81
        2             3.044944                         771.0           423.0     1.71
        3             3.595506                         804.0           445.0     1.80
```

```
4         -3.000000                    631.0        932.0    1.80

   magnesium  inorganicphosphorus
0       0.16                 2.86
1       0.15                 2.80
2       0.15                 2.81
3       0.15                 2.90
4       0.08                 2.64
```

Exploratory data Analysis

1. Check for the datatype for each of the variables

```
In [5]: #check for each column data type
        df.dtypes
```

```
Out[5]: cholinesterase                       float64
        triglyceride                         float64
        totalcholesterol                     float64
        highdensitylipoproteincholestero     float64
        lowdensitylipoproteincholesterol     float64
        glucose                              float64
        urea                                 float64
        immunoglobuling                      float64
        totalprotein                         float64
        albumin                              float64
        alkalinephosphatase                  float64
        hydroxybutyratedehydrogenase         float64
        creatinekinase                       float64
        calcium                              float64
        magnesium                            float64
        inorganicphosphorus                  float64
        dtype: object
```

```
In [6]: colnames=df.columns.values.tolist()
        colnames
```

```
Out[6]: ['cholinesterase',
         'triglyceride',
         'totalcholesterol',
         'highdensitylipoproteincholestero',
         'lowdensitylipoproteincholesterol',
         'glucose',
         'urea',
         'immunoglobuling',
         'totalprotein',
         'albumin',
         'alkalinephosphatase',
         'hydroxybutyratedehydrogenase',
```

```
 'creatinekinase',
 'calcium',
 'magnesium',
 'inorganicphosphorus']
```

CHECK FOR MISSING VALUES
Check for missing values and remove them if any

In [7]:
```
nullcheckdf=df.isnull()
nullcheckdf.head()
```

Out[7]:
```
   cholinesterase  triglyceride  totalcholesterol  \
0           False         False             False
1           False         False             False
2           False         False             False
3           False         False             False
4           False         False             False

   highdensitylipoproteincholestero  lowdensitylipoproteincholesterol  \
0                             False                             False
1                             False                             False
2                             False                             False
3                             False                             False
4                             False                             False

   glucose   urea  immunoglobuling  totalprotein  albumin  \
0    False  False            False         False    False
1    False  False            False         False    False
2    False  False            False         False    False
3    False  False            False         False    False
4    False  False            False         False    False

   alkalinephosphatase  hydroxybutyratedehydrogenase  creatinekinase  calcium  \
0                False                         False           False    False
1                False                         False           False    False
2                False                         False           False    False
3                False                         False           False    False
4                False                         False           False    False

   magnesium  inorganicphosphorus
0      False                False
1      False                False
2      False                False
3      False                False
4      False                False
```

In [8]:
```
print("True Mean there are Missing Values \n Falses Mean there are No Missing Values \n\
for v in zip(colnames):
    v=v[0]
```

```
            print(nullcheckdf[v].value_counts())
            print("--------------------------------------------")
            print()
```

```
True Mean there are Missing Values
 Falses Mean there are No Missing Values


False    89
Name: cholinesterase, dtype: int64
--------------------------------------------

False    89
Name: triglyceride, dtype: int64
--------------------------------------------

False    89
Name: totalcholesterol, dtype: int64
--------------------------------------------

False    89
Name: highdensitylipoproteincholestero, dtype: int64
--------------------------------------------

False    89
Name: lowdensitylipoproteincholesterol, dtype: int64
--------------------------------------------

False    89
Name: glucose, dtype: int64
--------------------------------------------

False    89
Name: urea, dtype: int64
--------------------------------------------

False    89
Name: immunoglobuling, dtype: int64
--------------------------------------------

False    89
Name: totalprotein, dtype: int64
--------------------------------------------

False    89
Name: albumin, dtype: int64
--------------------------------------------
```

```
False    89
Name: alkalinephosphatase, dtype: int64
--------------------------------------------

False    89
Name: hydroxybutyratedehydrogenase, dtype: int64
--------------------------------------------

False    89
Name: creatinekinase, dtype: int64
--------------------------------------------

False    89
Name: calcium, dtype: int64
--------------------------------------------

False    89
Name: magnesium, dtype: int64
--------------------------------------------

False    89
Name: inorganicphosphorus, dtype: int64
--------------------------------------------
```

Missing Values Analysis: From the above results. we see that there are no missing values.
DESCRIPTIVE STATISTICS

1. The Descriptive statistics for the numerical variables. The below show the following for each of the numerical variables.

   a) The number of entries/observation
   b) The Mean/Average for each of the variables entries
   c) std (standard deviation) of the values from the mean
   d) Quantiles: 25%, 50% and 75% quantiles
   e) Max(Maximum) and Min(Minimum) values

Python describe() function is used to produce the above stated statistics for all the numerical variables.

```
In [9]: df.describe()
```

```
Out[9]:        cholinesterase  triglyceride  totalcholesterol  \
        count       89.000000     89.000000         89.000000
        mean       345.725918      0.952921          0.918779
        std        151.563582      0.733795          0.398138
        min       -206.000000      0.010000          0.000000
        25%        272.000000      0.370000          0.660000
```

50%	332.000000	0.830000	0.860000
75%	428.000000	1.530000	1.140000
max	738.000000	2.920000	1.830000

	highdensitylipoproteincholestero	lowdensitylipoproteincholesterol \
count	89.000000	89.000000
mean	0.293596	0.240691
std	0.168032	0.106660
min	-0.010000	0.000000
25%	0.170000	0.160000
50%	0.240000	0.210000
75%	0.390000	0.290000
max	0.700000	0.500000

	glucose	urea	immunoglobuling	totalprotein	albumin \
count	89.000000	89.000000	89.000000	89.000000	8.900000e+01
mean	7.750449	8.134838	0.158633	37.307662	8.596100e+00
std	2.670234	2.889986	0.136992	10.187337	7.902623e+00
min	1.160000	-0.710000	0.000000	1.239213	4.080186e-07
25%	6.350000	6.020000	0.060000	31.830000	6.143622e-03
50%	8.430000	8.420000	0.130000	39.310001	1.160449e+01
75%	9.580000	10.110000	0.240000	44.040001	1.550000e+01
max	12.490000	13.420000	0.580000	65.459999	1.970000e+01

	alkalinephosphatase	hydroxybutyratedehydrogenase	creatinekinase \
count	89.000000	89.000000	89.000000
mean	1.823621	628.102765	628.954551
std	7.295374	150.669045	326.743981
min	-23.900000	19.146067	30.932585
25%	-2.000000	531.000000	422.000000
50%	1.000000	661.000000	534.000000
75%	7.000000	738.000000	799.000000
max	19.000000	907.000000	1589.000000

	calcium	magnesium	inorganicphosphorus
count	89.000000	89.000000	89.000000
mean	1.668090	0.057438	2.610337
std	0.122455	0.078197	1.000642
min	1.200000	-0.112360	1.230000
25%	1.600000	0.030000	2.010000
50%	1.640000	0.070000	2.500000
75%	1.770000	0.100000	3.010000
max	1.970000	0.220000	7.250000

2. The Descriptive statistics for the categorical variables.Below, the descriptive statistics for the categorical variables are produce using pandas **describe()** function with the include object parameter

   The statistics produced are:

a) **Count** -- The number of oberservations in each variable
b) **Unique** -- The the total number of unique entries for each variable
c) **top** -- The entry with the high frequency
d) **freq - Frequency** -- the total number the top value occurs in the variable

In [14]:
```
#import the matrix plotting library from the pandas library
from pandas.tools.plotting import scatter_matrix as sm
sm(df[0:4:1,4])
```

```
---------------------------------------------------------------------------

TypeError                                 Traceback (most recent call last)

<ipython-input-14-77a24c8d5341> in <module>()
      1 #import the matrix plotting library from the pandas library
      2 from pandas.tools.plotting import scatter_matrix as sm
----> 3 sm(df[0:4:1,4])


C:\Program Files (x86)\Microsoft Visual Studio\Shared\Anaconda3_64\lib\site-packages\pan
   2137             return self._getitem_multilevel(key)
   2138         else:
-> 2139             return self._getitem_column(key)
   2140
   2141     def _getitem_column(self, key):


C:\Program Files (x86)\Microsoft Visual Studio\Shared\Anaconda3_64\lib\site-packages\pan
   2144         # get column
   2145         if self.columns.is_unique:
-> 2146             return self._get_item_cache(key)
   2147
   2148         # duplicate columns & possible reduce dimensionality


C:\Program Files (x86)\Microsoft Visual Studio\Shared\Anaconda3_64\lib\site-packages\pan
   1838         """Return the cached item, item represents a label indexer."""
   1839         cache = self._item_cache
-> 1840         res = cache.get(item)
   1841         if res is None:
   1842             values = self._data.get(item)


TypeError: unhashable type: 'slice'
```
