# Supplementary material for: Investigation of biochemical and physiological parameters of the newborn Saiga antelope (Saiga tatarica) in Gansu Province, China
Source: PLoS One. 2019 Nov 26;14(11):e0224822. doi: 10.1371/journal.pone.0224822 (PMC6879164; doi:10.1371/journal.pone.0224822)
Supplement: S3 File — (PDF) [file pone.0224822.s003.pdf]

Python Analysis Notebook

November 18, 2018

In [1]:
```
#import the libreries for the analysis
import pandas as pd
import numpy as np
import matplotlib.pyplot as plt
%matplotlib inline
```

In [2]:
```
#read in the data from the csv file
df=pd.read_csv("mawoloCleanData.csv")
#view the first five rows from the dataset
df.head()
```

Out[2]:

	cholinesterase	triglyceride	totalcholesterol	\
0	266.0	1.16	1.83	
1	254.0	1.14	1.83	
2	214.0	1.48	1.77	
3	280.0	1.48	1.80	
4	535.0	0.33	0.60	

	highdensitylipoproteincholestero	lowdensitylipoproteincholesterol	\
0	0.69	0.48	
1	0.70	0.48	
2	0.62	0.48	
3	0.64	0.50	
4	0.17	0.16	

	glucose	urea	immunoglobuling	totalprotein	albumin	\
0	8.46	5.24	0.08	42.630001	4.080186e-07	
1	8.29	5.32	0.41	42.860001	2.040093e-06	
2	8.65	4.59	0.00	43.240002	6.097965e-06	
3	8.94	4.70	0.36	45.830002	1.421690e-05	
4	10.16	8.39	0.44	33.820000	2.841626e-05	

	alkalinephosphatase	hydroxybutyratedehydrogenase	creatinekinase	calcium	\
0	1.157303	784.0	686.0	1.74	
1	2.134831	774.0	694.0	1.81	
2	3.044944	771.0	423.0	1.71	
3	3.595506	804.0	445.0	1.80	

4	-3.000000	631.0	932.0	1.80

	magnesium	inorganicphosphorus
0	0.16	2.86
1	0.15	2.80
2	0.15	2.81
3	0.15	2.90
4	0.08	2.64

Exploratory data Analysis

1. Check for the datatype for each of the variables

In [3]:
```
#check for each column data type
df.dtypes
```

Out[3]:
```
                                    cholinesterase
float64 triglyceride                   float64
totalcholesterol                       float64
highdensitylipoproteincholestero       float64
lowdensitylipoproteincholesterol       float64
glucose                                float64
urea                                   float64
immunoglobuling                        float64
totalprotein                           float64
albumin                                float64
alkalinephosphatase                    float64
hydroxybutyratedehydrogenase           float64
creatinekinase                         float64
calcium                                float64
magnesium                              float64
inorganicphosphorus                    float64
dtype: object
```

In [4]:
```
colnames=df.columns.values.tolist()
colnames
```

Out[4]:
```
['cholinesterase',
 'triglyceride',
 'totalcholesterol',
 'highdensitylipoproteincholestero',
 'lowdensitylipoproteincholesterol',
 'glucose',
 'urea',
 'immunoglobuling',
 'totalprotein',
 'albumin',
 'alkalinephosphatase',
 'hydroxybutyratedehydrogenase',
```

```
'creatinekinase',
'calcium',
'magnesium',
'inorganicphosphorus']
```

CHECK FOR MISSING VALUES

Check for missing values and remove them if any

In [5]:
```
nullcheckdf=df.isnull()
nullcheckdf.head()
```

Out[5]:
```
   cholinesterase  triglyceride  totalcholesterol  \
0           False         False             False
1           False         False             False
2           False         False             False
3           False         False             False
4           False         False             False

   highdensitylipoproteincholestero  lowdensitylipoproteincholesterol  \
0                             False                             False
1                             False                             False
2                             False                             False
3                             False                             False
4                             False                             False

   glucose   urea  immunoglobuling  totalprotein  albumin  \
0    False  False            False         False    False
1    False  False            False         False    False
2    False  False            False         False    False
3    False  False            False         False    False
4    False  False            False         False    False

   alkalinephosphatase  hydroxybutyratedehydrogenase  creatinekinase  calcium  \
0                False                         False           False    False
1                False                         False           False    False
2                False                         False           False    False
3                False                         False           False    False
4                False                         False           False    False

   magnesium  inorganicphosphorus
0      False                False
1      False                False
2      False                False
3      False                False
4      False                False
```

In [6]:
```
#print("True Mean there are  Missing Values \n  Falses Mean there are  No Missing Values
#for v in zip(colnames):
#     v=v[0]
```

```
#       print(nullcheckdf[v].value_counts())
#       print("--------------------------------------------")
#       print()
```

Missing Values Analysis: From the above results. we see that there are no missing values.
DESCRIPTIVE STATISTICS

1. The Descriptive statistics for the numerical variables. The below show the following for each of the numerical variables.

   a) The number of entries/observation
   b) The Mean/Average for each of the variables entries c)
   std (standard deviation) of the values from the mean d)
   Quantiles: 25%, 50% and 75% quantiles
   e) Max(Maximum) and Min(Minimum) values

Python describe() function is used to produce the above stated statistics for all the numerical variables.

In [7]: df.describe()

Out[7]:

	cholinestera	triglyceri	totalcholestero
coun	89.0000	89.0000	89.00000
mean	345.7259	0.9529	0.91877
std	151.5635	0.7337	0.39813
min	-	0.0100	0.00000
25%	272.0000	0.3700	0.66000
50%	332.0000	0.8300	0.86000
75%	428.0000	1.5300	1.14000
max	738.0000	2.9200	1.83000
	00	00	0

	highdensitylipoproteincholestero	lowdensitylipoproteincholesterol	\
count	89.000000	89.000000	
mean	0.293596	0.240691	
std	0.168032	0.106660	
min	-0.010000	0.000000	
25%	0.170000	0.160000	
50%	0.240000	0.210000	
75%	0.390000	0.290000	
max	0.700000	0.500000	

	glucose	urea	immunoglobuling	totalprotein	albumin	\
count	89.000000	89.000000	89.000000	89.000000	8.900000e+01	
mean	7.750449	8.134838	0.158633	37.307662	8.596100e+00	
std	2.670234	2.889986	0.136992	10.187337	7.902623e+00	
min	1.160000	-0.710000	0.000000	1.239213	4.080186e-07	
25%	6.350000	6.020000	0.060000	31.830000	6.143622e-03	
50%	8.430000	8.420000	0.130000	39.310001	1.160449e+01	
75%	9.580000	10.110000	0.240000	44.040001	1.550000e+01	

max	12.490000	13.420000	0.580000	65.459999	1.970000e+01

	alkalinephosphatase	hydroxybutyratedehydrogenase	creatinekinase \
count	89.000000	89.000000	89.000000
mean	1.823621	628.102765	628.954551
std	7.295374	150.669045	326.743981
min	-23.900000	19.146067	30.932585
25%	-2.000000	531.000000	422.000000
50%	1.000000	661.000000	534.000000
75%	7.000000	738.000000	799.000000
max	19.000000	907.000000	1589.000000

	calcium	magnesium	inorganicphosphorus
count	89.000000	89.000000	89.000000
mean	1.668090	0.057438	2.610337
std	0.122455	0.078197	1.000642
min	1.200000	-0.112360	1.230000
25%	1.600000	0.030000	2.010000
50%	1.640000	0.070000	2.500000
75%	1.770000	0.100000	3.010000
max	1.970000	0.220000	7.250000

2. The Descriptive statistics for the categorical variables.Below, the descriptive statistics for the categorical variables are produce using pandas **describe()** function with the include object parameter

   The statistics produced are:

   a) **Count** -- The number of oberservations in each variable
   b) **Unique** -- The the total number of unique entries for each variable
   c) **top** -- The entry with the high frequency
   d) **freq - Frequency** -- the total number the top value occurs in the variable

In [8]:
```
reviewData=pd.read_csv("reviews Data.csv")
reviewData.head()
```

Out[8]:

	??	??.1	????	Total white	Lymphocte R	Intermediat	Granulocy	Lymphocy \
0	NaN	1	????	2.0	16.7	11.0	72.3	0.3
1	NaN	3	????	2.4	22.6	17.8	59.6	0.5
2	NaN	4	????	2.5	13.7	8.8	77.5	0.3
3	NaN	5	????	2.6	19.8	13.7	66.5	0.5
4	NaN	6	????	2.0	19.6	12.3	68.1	0.4

	# RB Cells	Hemoglobi	...	Hem. Contr.	RBC Dis. C. \
0	0.2	1.5	...	121.9	22.9
1	0.4	1.5	...	114.2	21.1
2	0.2	2.0	...	126.0	23.1
3	0.4	1.7	...	122.6	22.8
4	0.2	1.4	...	125.7	21.7

```
   RBC Dis. C..1  Total Num. of p.  Ave Plate Vol.  Platelet D.  Platelet P.  \
0            188              52.0            15.4         1258          8.1
1            185              46.5            14.7         1222          8.8
2            183              53.9            15.5         1184          7.5
3            186              52.0            15.3         1394          8.4
4            172              57.6            16.6         1353          7.7

   Platelet  L. CR  Platelet  P.  Platelet large cell ratio
0             4.6           1.1                        0.9
1             5.6           1.7                        1.2
2             4.6           0.8                        0.8
3             5.1           1.7                        1.3
4             4.8           1.4                        0.9

[5 rows x 23 columns]
```

In [9]:
```
col=reviewData.columns.values.tolist()
col
```

Out[9]:
```
['??',
 '??.1',
 '????',
 'Total white ',
 'Lymphocte R',
 'Intermediat',
 'Granulocy',
 'Lymphocy',
 '# RB Cells',
 'Hemoglobi',
 'Hemotocri',
 'Ave RB C.',
 'Hemob. con',
 'Hem. Contr.',
 'RBC Dis. C.',
 'RBC Dis. C..1',
 'Total Num. of p.',
 'Ave Plate Vol.',
 'Platelet D.',
 'Platelet P. ',
 'Platelet  L. CR',
 'Platelet  P.',
 'Platelet large cell ratio']
```

In [12]:
```
reviewData.drop(columns=['??','??.1','????'],inplace=True)
reviewData.head()
```

Out[12]:
```
   Total white   Lymphocte R  Intermediat  Granulocy  Lymphocy  # RB Cells  \
0          2.0          16.7         11.0       72.3       0.3         0.2
```

```
1          2.4        22.6        17.8        59.6         0.5          0.4
2          2.5        13.7         8.8        77.5         0.3          0.2
3          2.6        19.8        13.7        66.5         0.5          0.4
4          2.0        19.6        12.3        68.1         0.4          0.2

   Hemoglobi  Hemotocri  Ave RB C.  Hemob. con  Hem. Contr.  RBC Dis. C.  \
0        1.5       6.88        158        83.8        121.9         22.9
1        1.5       6.76        143        77.1        114.2         21.1
2        2.0       6.82        158        85.9        126.0         23.1
3        1.7       6.82        156        83.6        122.6         22.8
4        1.4       6.81        148        85.6        125.7         21.7

   RBC Dis. C..1  Total Num. of p.  Ave Plate Vol.  Platelet D.  Platelet P.   \
0            188              52.0            15.4         1258           8.1
1            185              46.5            14.7         1222           8.8
2            183              53.9            15.5         1184           7.5
3            186              52.0            15.3         1394           8.4
4            172              57.6            16.6         1353           7.7

   Platelet  L. CR  Platelet  P.  Platelet large cell ratio
0              4.6          1.01                        0.9
1              5.6          1.07                        1.2
2              4.6          0.88                        0.8
3              5.1          1.17                        1.3
4              4.8          1.04                        0.9
```

In [13]:
```
reviewData.dtypes
```

Out[13]:
```
Total white                  float64
Lymphocte R                  float64
Intermediat                  float64
Granulocy                    float64
Lymphocy                     float64
# RB Cells                   float64
Hemoglobi                    float64
Hemotocri                    float64
Ave RB C.                      int64
Hemob. con                   float64
Hem. Contr.                  float64
RBC Dis. C.                  float64
RBC Dis. C..1                  int64
Total Num. of p.             float64
Ave Plate Vol.               float64
Platelet D.                    int64
Platelet P.                  float64
Platelet  L. CR              float64
Platelet  P.                 float64
Platelet large cell ratio
float64 dtype: object
```

In [25]:
```
ndata=reviewData[['Total white ','Lymphocte R','Intermediat','Granulocy']]
ndata.head()
```

Out[25]:

	Total white	Lymphocte R	Intermediat	Granulocy
0	2.0	16.7	11.0	72.3
1	2.4	22.6	17.8	59.6
2	2.5	13.7	8.8	77.5
3	2.6	19.8	13.7	66.5
4	2.0	19.6	12.3	68.1

In [29]:
```
ndata[['cholinesterase','triglyceride','totalcholesterol','highdensitylipoproteinchol
ndata.head()
```

```
C:\Program Files (x86)\Microsoft Visual Studio\Shared\Anaconda3_64\lib\site-packages\pandas\co
A value is trying to be set on a copy of a slice from a DataFrame.
Try using .loc[row_indexer,col_indexer] = value instead

See the caveats in the documentation: http://pandas.pydata.org/pandas-docs/stable/indexing.htm
  self[k1] = value[k2]
```

Out[29]:

	Total white	Lymphocte R	Intermediat	Granulocy	cholinesterase \
0	2.0	16.7	11.0	72.3	266.0
1	2.4	22.6	17.8	59.6	254.0
2	2.5	13.7	8.8	77.5	214.0
3	2.6	19.8	13.7	66.5	280.0
4	2.0	19.6	12.3	68.1	535.0

	triglyceride	totalcholesterol	highdensitylipoproteincholestero
0	1.16	1.83	0.69
1	1.14	1.83	0.70
2	1.48	1.77	0.62
3	1.48	1.80	0.64
4	0.33	0.60	0.17

In [35]:
```
ndata.plot(kind='Line',legend=True,figsize=(14,8),grid=True)
```

Out[35]: <matplotlib.axes._subplots.AxesSubplot at 0x1698473b0b8>

In [36]: ndata.describe()

Out[36]:

	Total white	Lymphocte R	Intermediat	Granulocy	cholinesterase	\
count	40.000000	40.000000	40.000000	40.000000	40.000000	
mean	2.462500	18.592500	16.495000	64.907500	338.290169	
std	0.848434	3.874002	8.120438	10.037274	111.665106	
min	1.100000	12.100000	7.700000	42.200000	-22.000000	
25%	1.975000	16.275000	11.175000	59.525000	276.500000	
50%	2.500000	17.950000	13.300000	67.650000	334.000000	
75%	2.850000	20.700000	18.075000	71.900000	424.750000	
max	4.500000	29.800000	35.100000	79.100000	535.000000	

	triglyceride	totalcholesterol	highdensitylipoproteincholestero
count	40.000000	40.00000	40.000000
mean	0.798000	0.90225	0.282750
std	0.741627	0.46739	0.187165
min	0.010000	0.00000	-0.010000
25%	0.217500	0.59750	0.170000
50%	0.465000	0.72000	0.190000
75%	1.240000	1.25250	0.432500
max	2.800000	1.83000	0.700000

In [74]:
```
plt.style.use("ggplot")
plt.figure(figsize=(8,12))
plt.title("Comparative analysis of Total White \& cholinesterase")
plt.subplot(2, 1, 1)
plt.hist(ndata['Total white '], orientation='horizontal',histtype='bar')
```

```
plt.ylabel("Total  White")
plt.xlabel("frequencies")
plt.subplot(2, 1, 2)
plt.hist(ndata['cholinesterase'],orientation='horizontal')
plt.xlabel("cholinesterase")
plt.ylabel("frequencies")
plt.show()
```
