# Supplementary material for: Investigation of biochemical and physiological parameters of the newborn Saiga antelope (Saiga tatarica) in Gansu Province, China
Source: PLoS One. 2019 Nov 26;14(11):e0224822. doi: 10.1371/journal.pone.0224822 (PMC6879164; doi:10.1371/journal.pone.0224822)
Supplement: S6 File — (PDF) [file pone.0224822.s006.pdf]

MATERIALS AND METHODS PROTOCOL PROCEDURES

Spectrophotometry method for the detection of Biochemical parameters

Definition of key terms

Spectrophotometry is an experimental technique that is used to measure the concentration of solutes in a specific solution by calculating the amount of light absorbed by those solutes.[1] This technique is powerful because certain compounds will absorb different wavelengths of light at different intensities. By analyzing the light that passes through the solution, you can identify particular dissolved substances in solution and how concentrated those substances are. A automatic analyser (7180) is the device used to analyze solutions in a laboratory research setting.

Desecription of Automatic analyser

The Automatic analyser (7180) is a medium-sized biochemistry automatic analyzer.The minimum required amount of reaction solution is smaller due to a new optical design, and running cost is lower and working efficiency higher because the operating unit is usable in the same way as a general-purpose personal computer. Further, this analyzer features a wide range of applications including routine, stat and special analyses.

Operating Unit

The analyzer is now easier to operate than before because of its user interface featuring an operability unique to a general purpose OS (Windows®) and the touch screen.

Analytical Unit

After deliberation on how best to make opera-tions comfortable and analysis smooth, the Model 7180 was developed. Its operator-friendly design concept is visible everywhere from the action warning lamp to the transparent top cover which allows checking of the disks at any time. With this, we started the manchine and its ran the entire tests and we obtained our sample results.

Routine Test

The 7180 has a high capability of simultaneous 86 channels and 800 tests/hour adequate as a routine analyzer and has a flexibility in application to new tests and new assays.The enriched flexible random access function wider in degree of freedom has raised the potentiality of clinical tests to an even higher level.

Basically, there are three (3) major parts involve in using this method with series of steps. The below are detail procedures:

PART I: Sample preparation

1. **Turning on the automatic analyser:** The **automatic analyser** was turned on to warm up to give an accurate reading.The warm up process toke fifteen (15) minutes before we run our samples.

While the **automatic analyser** was warming up, we prepare our samples to be run.

2. **Loading of the proper volume of the sample into the cuvette:** The automatic analyser contains a volume of 1 milliliter (mL) and test tubes with a volume of 5 mL.

- different pipettes were used for each sample to prevent contamination. [3]

3. **Preparation of a control solution:** Known as a blank, our control solution has chemical solvent in which the solute to be analyzed is dissolved in. The blank is the same volume as the solution to be analyzed and kept in the same kind of container.

4. **Wipeing the outside of the cuvette:** Before placing our cuvette into the automatic analyser, we make sure that the cuvette were cleaned to avoid interference from dirt or dust particles. We used a lint free cloth, and remove water droplets or dust that were on the outside of the cuvette.[4]

PART II: Running the experiment

1. **Selected and set the wavelength of light to analyze the sample with:** We used a single wavelength of light (monochromatic color) to make the testing more effective. The color of the light chosen were the one known to be absorbed by one of the chemicals thought to be in the test solute. We set the desired wavelength according to the specifications of your spectrophotometer.

2. **Calibrate the machine with the blank: We** Placed the blank into the cuvette holder and shuted the lid. On an analog automatic analyser, there was a screen with a needle that moves based on the intensity of light detection. When the blank got in, we saw the needle moved to the right. The values were recorded, while the blank still in the machine, we moved the needle to zero using the adjustment knob.

   - When we removed the blank, the calibration was still in place. During measuring the rest of our samples, the absorbance from the blank was automatically subtracted out.
   - A single blank was use per session so that each sample is calibrated to the same blank.

3. **The blank were removed and calibration tested:** We removed the blank and the needle should stayed at 0 (zero). The blank was back into the machine and ensured the needle readout doesn't change. If the machine is properly calibrated with your blank, everything should stay at 0.

4. **Measurement of the absorbance of experimental sample:** We removed the blank and placed the experimental sample into the machine. We slide the cuvette into the designated groove and ensure it stond upright. We waited about 10 seconds until the needle is steady or until the digital numbers changed. We recorded the values of % transmittance and/or absorbance.

   - The absorbance is also known as the optical density (OD).
   - The more light that is transmitted, the less light the sample absorbs. Generally, we wanted to record the absorbance values which will usually be given as a decimal, for example, 0.43.

5. **Repeated the test with successive wavelengths of light.** Our samples have multiple unknown compounds that vary in their absorbance

depending on wavelength. To eliminate uncertainty, we repeated our readings at 25 nm intervals across the spectrum. This allow us to detect other chemicals suspected to be in the solute.

PART III: Analyzing the Absorbance Data

1. **Calculation of the transmittance and absorbance of the sample:** Transmittance is how much of the light that passed through the sample reached the automatic analyser. Absorbance is how much of the light has been absorbed by one of the chemicals in the solute. Many modern automatic analyser have an output of transmittance and absorbance, when we recorded the intensity, we calculated these values.[5]

- The transmittance (T) is found by dividing the intensity of the light that passed through the sample solution with the amount that passed through the blank. It is normally expressed as a decimal or percentage. $T = I/I_0$ where I is the intensity of the sample and I_0 is the intensity of the blank.
- The absorbance (A) is expressed as the negative of the base-10 logarithm (exponent) of the transmittance value: $A = -\log_{10}T$.[6] For a T value of 0.1, the value of A is 1 (0.1 is 10 to the -1 power), meaning 10% of the light is transmitted and 90% is absorbed. For a T value of 0.01, the value of A is 2 (0.01 is 10 to the -2 power), meaning 1% of the light is transmitted.

2. **Plotting the absorbance values versus the wavelengths on a graph.** The absorbance value is plotted on the vertical y-axis against the wavelength of light used for a given test plotted on the horizontal x-axis. Plotting the maximum absorbance values for each wavelength of light tested, produces the sample's absorbance spectrum and identifies the compounds making up the test substance and their proportions.

- An absorbance spectrum usually has peaks at certain wavelengths that can allow you to identify specific compounds.

3. **Comparing our absorbance spectrum plot to known plots of specific compounds:** Compounds have unique absorbance spectrum and always produced a peak at the same wavelength every time they are

measured. Comparing our plots of unknown compounds to those of known compounds, we identified the solutes that compose of our solution.

- This method can also to identify contaminants in sample. If you are expecting 1 clear peak at a specific wavelength and you get 2 peaks at separate wavelengths, you know something is not right in your sample.

Materials Required:

1. Automatic analyser
2. Cuvette
3. Blank solution
4. Glass
5. Wash bottle
6. Lint free cloth
7. blank

Reagents:

1. Cobalt (II) chloride
2. Hexaaquacobalt (II) ion
3. Ferrocene
4. Crystal violet
5. Rose bengal
6. Coumarin

Safety methods observed during our experiment at the laboratory:

1. We wore lab coat and gloves while in the lab. When we enter the lab, exhausted fans were switch on and we make sure that all the chemicals and reagents required for the experiment were available.

2. All working apparatus were cleaned with chromic acid and distilled water and we ensure that all the apparatus are free from water droplets while performing the experiment.
3. We calibrated the electronic weigh balance before taking the measurements.
4. Ensuring that the spectrophotometer working properly was done.
5. We make sure the cuvette was handle with tissue paper and didn't touch it with our hands.
6. The cuvette was wipe with tissue paper before placing in the spectrophotometer.
7. All apparatus were cleaned with soap and distilled water. Upon completion of the experiment, we recap the reagent bottles. Switch off the light and exhaust fan before leaving the lab.
8. Discarded the used gloves in a waste bin.

Benefit of Spectrophotometry method

The usage of Automatic analyser (Hitachi Ltd 7180 Serial, Tokyo, Japan) enabled us to obtained more samples result in less time and this prevented other factors from influencing our tested results.

References:

1. http://www2.bren.ucsb.edu/~keller/courses/esm223/Spectrometer_analysis.pdf
2. http://www2.bren.ucsb.edu/~keller/courses/esm223/Spectrometer_analysis.pdf
3. http://www2.bren.ucsb.edu/~keller/courses/esm223/Spectrometer_analysis.pdf
4. http://www2.bren.ucsb.edu/~keller/courses/esm223/Spectrometer_analysis.pdf
5. http://www.chm.davidson.edu/vce/spectrophotometry/Spectrophotometry.html
6. http://www.chm.davidson.edu/vce/spectrophotometry/Spectrophotometry.html

Coulter Method for the detection of Physiological parameters

Instrument

A **Coulter counter** [1] [2] is an apparatus for counting and sizing particles suspended in electrolytes. It is used for cells, bacteria, prokaryotic cells and virus particles.[3]

A typical Coulter counter has one or more microchannels that separate two chambers containing electrolyte solutions. As fluid containing particles or cells is drawn through each microchannel, each particle causes a brief change to the electrical resistance of the liquid. The counter detects these changes in electrical resistance.

Coulter principle

The *Coulter principle* states that particles pulled through an orifice, concurrent with an electric current, produce a change in impedance that is proportional to the volume of the particle traversing the orifice. This pulse in impedance originates from the displacement of electrolyte caused by the particle. The Coulter principle was named for its inventor, Wallace H. Coulter. The principle has found commercial success in the medical industry, particularly in hematology, where it can be applied to count and size the various cells that make up whole blood.

Cells, being poorly conductive particles, alter the effective cross-section of the conductive microchannel. If these particles are less conductive than the surrounding liquid medium, the electrical resistance across the channel increases, causing the electric current passing across the channel to briefly decrease. By monitoring such pulses in electric current, the number of particles for a given volume of fluid can be counted. The size of the electric current change is related to the size of the particle, enabling a particle size distribution to be measured, which can be correlated to mobility, surface charge, and concentration of the particles.

The Coulter Counter is a vital constituent of today's hospital laboratory. Its primary function being the quick and accurate analysis of complete blood counts (often referred to as CBC). The CBC is used to determine the number or proportion of white and red blood cells in the body. Previously, this procedure involved preparing a blood cell stain and manually counting each type of cell under a microscope, a process that typically took a half-hour.

Coulter Counters have a wide variety of applications including paint, ceramics, glass, molten metals and food manufacture. They are also routinely employed for quality control.

A Coulter counter played an important role in the development of the first ever cell sorter, and was involved in the early days of the development of flow cytometry. Even today, some flow cytometers utilize the Coulter Principle to provide highly accurate information about cell size and count.

Many investigators have designed a variety of devices based on the Coulter Principle, and generated peer-reviewed publications featuring data generated by these devices. A few of these devices have also been commercialized. All implementations of the Coulter Principle feature trade offs between sensitivity, noise shielding, solvent compatibility, speed of measurement, sample volume, dynamic range, and reliability of device manufacture.

Development

Wallace H Coulter discovered the Coulter Principle in the late 1940s (though a patent was not awarded until October 20, 1953). Coulter was influenced by the atomic bombs dropped on Hiroshima and Nagasaki. These events motivated Coulter to simplify and improve blood cell analysis so that large populations could be screened rapidly, as would be necessary in the event of a nuclear war. Partial funding of the project came from a grant award from the Office of Naval Research.[4][5]

"Coulter Principle" refers to the use of an electric field for counting and sizing dilute suspensions of particles in conducting liquids. Wallace H. Coulter was awarded US Patent #2,656,508, *Means for Counting Particles Suspended in a Fluid*. The Coulter Principle is most commonly employed in a Coulter counter, which is an analytical instrument designed for a specific task such as counting cells. However, there are numerous other ways to implement the Coulter Principle. Several of these have been attempted, some with commercial success, and some purely for academic research. To date, the most commercially successful application of the Coulter Principle is in hematology, where it is used to obtain information about patients' blood cells.

The Coulter Principle relies on the fact that particles moving in an electric field cause measurable disturbances in that field. The magnitudes of these disturbances are proportional to the size of the particles in the field. Coulter identified several requirements necessary for practical application of this phenomenon. First, the particles should be suspended in a conducting liquid. Second, the electrical field should be physically constricted so that the movement of particles in the field causes detectable changes in the current. Finally, the particles should be dilute enough so

that only one at a time passes through the physical constriction, preventing an artifact known as coincidence.

While the Coulter Principle can be implemented in a variety of designs, there are two that have become the most commercially relevant. These include an aperture format and a flow cell format.

Experimental Procedures

Coincidence

Anomalous electrical pulses was generated because the concentration of samples were so high that multiple particles entered the aperture simultaneously. This situation is known as coincidence. This condition occured because there was almost no way to ensure that a single large pulse is the result of a single large particle or multiple small particles entering the aperture at once. To prevent this situation, samples must be fairly dilute, but we didn't dilute our samples because we needed an accurate results.

Particle path

The shape of the generated electrical pulse varied with the particle path through the aperture. Shoulders and other artifacts occured because the electric field density varied across the diameter of the aperture. This variance is a result of both the physical constriction of the electric field and also the fact that the liquid velocity varied as a function of radial location in the aperture. In the flow cell format, this effect was minimized since sheath flow ensured each particle traveled an almost identical path through the flow cell. In the aperture format, we used the signal processing algorithms to correct the artifacts resulting from particle path.

Conductive particles

Conductive particles are often common concern for considering the Coulter Principle. Although, there are interesting scientific questions, on this procedure, it rarely affects the results of an experiment. This is because the conductivity difference between most conductive materials and ions in liquid (referred to as the discharge potential) is so great that most conductive materials act as insulators in a Coulter counter. The voltage we used to break down this potential barrier is referred to as the breakdown voltage. For those highly conductive materials that presented problems, the voltage we used during our Coulter experiment reduced below the breakdown potential (which was determined empirically).

Porous particles

We use the Coulter principle to measured the volume of our samples, since the disturbance in the electric field is proportional to the volume of electrolyte displaced from the aperture. This medium has led to confusion amongst researchers who are use to optical measurements from microscopes or other systems that only view two dimensions and also show the boundaries of a sample. The Coulter Principle, on the other hand measured three dimensions and the volume displaced by a sample.

Direct current vs alternating current

Direct current has been used in the Coulter counters found in most research and cell laboratories. Direct current measurements are useful for an array of particles and allow for simplified data acquisition and processing. Base on this, we used direct current to simplified and processed data acquisition. Alternating current measurements are sometimes used in clinical hematology instruments, due to the special nature of cell membranes. At low frequencies (below 500 kHz), alternating and direct current measurements behave essentially the same way. At intermediate frequencies (500 kHz - 6 MHz), the plasma membrane of cells can become polarized, leading to a decreased capacitance of the measurement systems. However, at high frequencies (6-20 MHz), the cell membrane loses its polarization, and the electrical pulses provide information about the cell cytoplasm.

Major applications

Hematology/Physiology

The most successful and important application of the Coulter Principle is in the characterization of blood cells. The technique has been used to diagnose a variety of diseases, and is the standard method for obtaining red blood cell counts (RBCs) and white blood cell counts (WBCs) as well as several other common parameters. When combined with other technologies such as fluorescence tagging and light scattering, the Coulter Principle can help produce a detailed profile of patients' blood cells.

Cell count and size

In addition to clinical counting of blood cells (cell diameters of ~6-10 micrometres, typically), the Coulter principle has established itself as the most reliable laboratory method for counting a wide variety of cells, ranging from bacteria (< 1 micrometre in size), fat cells (~400 micrometre), plant cell aggregates (>~1200 micrometre), and stem cell embryoid bodies (~900 micrometre). The technique has become so standardized that ASTM International has published a procedure on the topic: *ASTMF2149-01(2007) Standard Test Method for Automated Analyses of Cells-the Electrical Sensing Zone Method of Enumerating and Sizing Single Cell Suspensions*.

Particle characterization

The Coulter Method has proved useful for applications well beyond cellular studies. The fact that it individually measures particles, is independent of any optical properties, is extremely sensitive, and is very reproducible has appeal to a wide variety of fields. Consequently, the Coulter Principle has been adapted to the nanoscale to produce a novel nanoparticle characterization technique called Tunable Resistive Pulse Sensing, or TRPS. TRPS enables high-fidelity analysis of a diverse set of nanoparticles, including (but not limited to): functionalized drug delivery nanoparticles, Virus-like particles (VLPs), liposomes, exosomes, polymeric nanoparticles, microbubbles.

Safety methods observed during our experiment at the laboratory:

1. We wore lab coat and gloves while in the lab. When you enter the lab, exhausted fans were switch on and we make sure that all the chemicals and reagents required for the experiment were available.

2. All working apparatus were cleaned with chromic acid and distilled water and we ensure that all the apparatus are free from water droplets while performing the experiment.

3. We calibrated the electronic weigh balance before taking the measurements.

4. Ensuring that the spectrophotometer working properly was done.

5. We make sure the cuvette was handle with tissue paper and didn't touch it with our hands.

6. The cuvette was wipe with tissue paper before placing in the spectrophotometer.

7. All glassware were cleaned with soap and distilled water. Upon completion of the experiment, we recap the reagent bottles. Switch off the light and exhaust fan before leaving the lab.

8. Discarded the used gloves in a waste bin.

Benefits of Coulter Method

- Increase productivity with consistently reliable results.

- With its versatile closed tube sampling system, the coulter save time and enhance safety for laboratorians.

- Flexible specially-formulated reagents, fully automated QC and calibration platforms provide consistently reliable results.

References

1. W.R. Hogg, W. Coulter; Apparatus and method for measuring a dividing particle size of a particulate system; United States Patent 3557352

2. U.S. Patent 7,397,232 *Coulter counter*

3. *R.W. DeBlois, C.P. Bean (1970). "Counting and sizing of submicron particles by the resistive pulse technique". Review of Scientific Instruments.* ***41*** *(7): 909–916.*

4. Bibcode*:*1970RScI...41..909D. doi*:*10.1063/1.1684724.

   Marshall Don. Graham (2003). "The Coulter Principle: Foundation of an Industry". Journal of Laboratory Automation. ***8*** *(6): 72–81.*

5. *Cytometry volume 10*, a DVD series produced by the Purdue University Cytometry Labs http://www.cyto.purdue.edu/cdroms/cyto10a/seminalcontributions/coulter.html
