# Supplementary material for: Investigation of biochemical and physiological parameters of the newborn Saiga antelope (Saiga tatarica) in Gansu Province, China
Source: PLoS One. 2019 Nov 26;14(11):e0224822. doi: 10.1371/journal.pone.0224822 (PMC6879164; doi:10.1371/journal.pone.0224822)
Supplement: S7 File — (PDF) [file pone.0224822.s007.pdf]

BIOMEDICAL INDEX STATISTICS

		Female		Male		
	Variable	Sample Size	Mean ± SD	Sample Size	Mean ± SD	independent t-test P-Value
1	Cholinesterase	50	36.66 ± 34.332	36	52.056 ± 58.931	> 0.1311
2	Triglyceride	50	0.76 ± 0.483	36	1.238 ± 0.914	**< **0.0024**
3	Total cholesterol	50	0.89 ± 0.345	36	1.027 ± 0.413	> 0.0888
4	High density lipoprotein cholesterol	50	0.27 ± 0.154	36	0.338 ± 0.176	> 0.0766
5	Low density lipoprotein cholesterol	50	0.23 ± 0.095	36	0.276 ± 0.108	**< *0.0241**
6	glucose	50	8.24 ± 2.156	36	7.446 ± 2.916	> 0.1522
7	Urea	50	8.73 ± 2.693	36	7.964 ± 2.206	> 0.1628
8	Immunoglobulin G	50	0.15 ± 0.145	36	0.178 ± 0.127	> 0.3752
9	Total protein	50	39.66 ± 16.246	36	39.218 ± 8.123	> 0.8818
10	albumin	50	24.60 ± 6.246	36	23.633 ± 6.676	> 0.4943
11	Alkaline phosphatase	50	5.22 ± 16.621	36	31.167 ± 136.371	> 0.1856
12	Hydroxybutyrate dehydrogenase	50	652.46 ± 196.890	36	631.806 ± 136.998	> 0.5895
13	Creatine kinase	50	740.56 ± 348.729	36	501.639 ± 225.249	**< ***0.00053**
14	calcium	50	-1.67 ± 0.098	36	-1.673 ± 0.135	> 0.9476
15	magnesium	50	0.05 ± 0.084	36	-0.203 ± 1.681	> 0.2858
16	Inorganic phosphoru	50	2.54 ± 1.141	36	2.629 ± 0.632	> 0.6707

P-VALUE MARKED WITH * ARE SIGNIFICANT
