# Supplementary material for: Investigation of biochemical and physiological parameters of the newborn Saiga antelope (Saiga tatarica) in Gansu Province, China
Source: PLoS One. 2019 Nov 26;14(11):e0224822. doi: 10.1371/journal.pone.0224822 (PMC6879164; doi:10.1371/journal.pone.0224822)
Supplement: S8 File — (PDF) [file pone.0224822.s008.pdf]

	Variable	Female Sample Size	Female Mean ± SD	Male Sample Size	Male Mean ± SD	t-test P-Value
1	Total white blood cells	18	2.48 ± 0.91	21	2.38 ± 0.78	> 0.7067
2	Lymphocyte ratio	18	19.51 ± 4.46	21	18.03 ± 3.19	> 0.2386
3	Intermediate cell ratio	18	16.15 ± 8.12	21	17.21 ± 8.25	> 0.6894
4	Granulocyte ratio	18	64.33 ± 10.12	21	64.76 ± 10.00	> 0.8963
5	Lymphocyte	18	0.49 ± 0.24	21	0.41 ± 0.13	> 0.1915
6	Intermediate cell	18	0.36 ± 0.17	21	0.39 ± 0.24	> 0.6628
7	Granulocytes	18	1.63 ± 0.67	21	1.58 ± 0.60	> 0.7987
8	Total number of red blood cells	18	6.60 ± 1.70	21	6.93 ± 1.16	> 0.4765
9	Hemoglobin	18	151.33 ± 42.63	21	157.43 ± 30.51	> 0.6070
10	Hematocrit	18	73.59 ± 13.32	21	79.49 ± 8.86	> 0.1073
11	Average red blood cell volume	18	123.42 ± 3.80	21	120.82 ± 3.28	**< 0.0277**
12	Hemoglobin content	18	22.74 ± 1.03	21	22.59 ± 0.88	> 0.6293
13	Hemoglobin concentration	18	182.33 ± 8.11	21	184.76 ± 6.80	> 0.3154
14	Red blood cell distribution width SD	18	52.97 ± 3.09	21	53.00 ± 3.40	> 0.9754
15	Red blood cell distribution width CV	18	15.56 ± 0.69	21	15.84 ± 0.87	> 0.2667
16	Total number of platelets	18	1089.72 ± 282.94	21	1203.14 ± 205.20	> 0.1563
17	Average platelet volume	18	8.02 ± 0.46	21	8.29 ± 0.54	> 0.0986
18	Platelet distribution width	18	5.02 ± 0.83	21	4.97 ± 0.34	> 0.7810
19	Platelet pressure	18	0.88 ± 0.26	21	1.00 ± 0.19	> 0.1021
20	Platelet large cell ratio	18	0.92 ± 0.17	21	0.99 ± 0.19	> 0.2461
